# Living Kombucha Electronics with Proteinoids

**DOI:** 10.1021/acsomega.4c09743

**Published:** 2025-05-20

**Authors:** Anna Nikolaidou, Panagiotis Mougkogiannis, Andrew Adamatzky

**Affiliations:** † Unconventional Computing Laboratory, 1981 University of the West of England, Bristol BS16 1QY, U.K.; ‡ School of Architecture and Environment, University of the West of England, Coldharbour Ln, Stoke Gifford, Bristol BS16 1QY, U.K.

## Abstract

The work introduces a composite material that combines Kombucha cellulose mats with synthetic thermal proteinoids to create electroactive biofilms, capable of sensing and computation. The synthesis of proteinoids involves heating amino acid mixtures, which leads to the formation of proto–cell structures capable of biological electrical signaling. We demonstrate that these hybrid biofilms exhibit adjustable memristive and memfractance properties, which can be utilized for unconventional computing tasks. The potential applications of living biofilms extend beyond neural interfaces, encompassing bioinspired robotics, smart wearables, adaptive biorobotic systems, and other technologies that rely on dynamic bioelectronic materials. The composite films offer a wide range of options for synthesis and performance customization. Current research is dedicated to customizing the composition, nanostructure, and integration of proteinoids in hybrid circuits to achieve specific electronic functionalities. Overall, these cross–kingdom biofilms are an intriguing category of materials that combine the unique properties of biological organisms and smart polymers. The Kombucha–proteinoid composites are a significant step forward in the development of future technologies that bridge the gap between living and artificial life systems. These composites have the remarkable ability to support cellular systems and demonstrate adaptive bioelectronic behavior.

## Graphical abstract

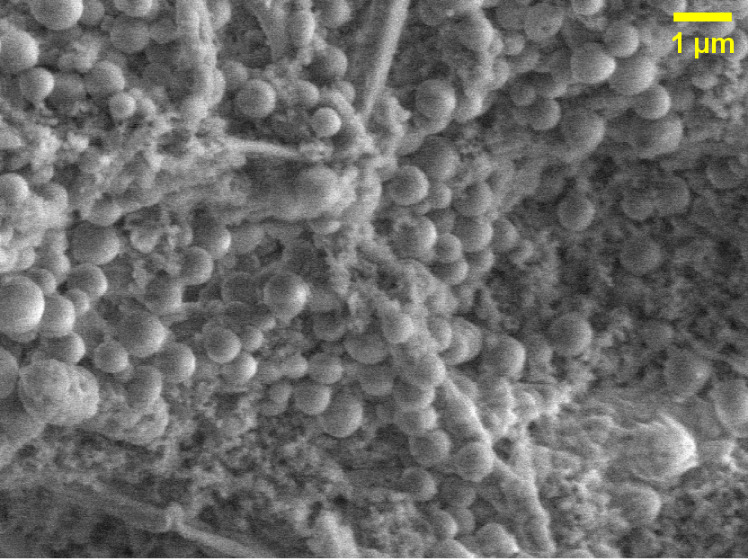

## Introduction

### Origins and Evolution of Kombucha

Kombucha is a fermented tea beverage reported to have originated in Northeast China in around 220 BC and consumed extensively during the Qin Dynasty.^1^ The fermented tea, referred to as “Manchurian mushroom” or “kombu tea”,^2,3^ was introduced to Japan in approximately 414 CE and employed for the purpose of alleviating Emperor Inkyo’s gastrointestinal ailments.^4^ Kombucha spread over trade channels and eventually reached Russia and Eastern Europe during the next centuries.^4^

Germany saw the introduction of Kombucha in the early 20th century. World War II facilitated the widespread introduction of it in Europe, leading to its increased popularity in France and North Africa due to its advantageous effects on health.^5,6^ Nevertheless, the scarcity of tea and sugar during wartime resulted in the loss of its popularity.^7^ In the 1950s, Swiss scientists sparked renewed interest by comparing Kombucha to yogurt in terms of their ability to enhance gut microorganisms.^8^

In the present day, the process of making Kombucha involves soaking tea and sugar, allowing it to cool, and then introducing a symbiotic culture of bacteria and yeast known as SCOBY.^1^ The culture undergoes aerobic fermentation for a duration ranging from several days to weeks, resulting in the formation of cellulosic films. The exact composition of the SCOBY, the type and concentration of tea and sugar used,^9,10^ the oxygen concentrations, fermentation time,^11,12^ and temperature^11,13^ can affect the composition of the Kombucha cellulose films. For the production of the Kombucha beverage, the completed Kombucha undergoes filtration, infusion of flavors, and is subsequently stored in a refrigerated environment.^4^ Currently, the beverage, SCOBY cultures, and preparation kits are readily accessible for purchase in the market.

### Bridging Prebiotic Conditions to Cellular Life: Proteinoids

Proteinoids, which were initially characterized by Sidney Fox in 1959, are abiotic synthetic polypeptides.^14,15^ These are produced by heating mixtures of amino acids to trigger polymerization. The self-assembly of proteinoids, which are rich in glutamic and aspartic acid, leads to the formation of proteinoid microspheres.

The proteinoids microspheres demonstrate compartmentalization, selective permeability, and other lifelike qualities that resemble protocells. Their formation from easily accessible precursors supports models of early biological evolution progressing from simple to complex polymers. Proteinoids have a unique quality of being formed solely from amino acid precursors, setting them apart from other protocell models. Chemical and structural characterizations of proteinoids have been conducted in previous studies.^16–21^ The covalent bonds formed between the building blocks by heating mixtures of amino acids generate a three-dimensional network structure that resembles the protein structures observed in nature.^22^ The microspherical aggregates of proteinoids, formed from many individual proteinoid particles when immersed in solution, are typically hollow with a homogeneous and smooth surface texture.^22,23^ Different solution conditions impact the morphology and assembly processes of the proteinoids microstructure, affecting their electrochemical characteristics. The presence of delocalized electrons that move freely within the material network, allow the flow of electric current and enable the proteinoid network to conduct electricity. The conductivity and electrical properties are therefore determined by their composition and microstructure.^20,22^

In addition to microspheres, proteinoids exhibit additional biomimetic capabilities such as catalytic activity, microtubule formation, and membrane generation.^24–27^ Despite the changing environmental conditions on primitive Earth, their ability to self-organize in an orderly manner remained unaffected. Proteinoids provide a strong connection between prebiotic chemistry and the earliest minimal cells.

The study of proteinoids offers valuable insights into potential pathways for the origins of life that cannot be obtained solely through modern biochemistry.^28^ Their synthesis involves using thermal energy to produce organized biomolecular systems. The protocells that have been proposed function as photon absorbers, thereby increasing the production of entropy. Proteinoids offer a thermodynamically feasible pathway to cellular structures that are compatible with life, taking advantage of nonequilibrium conditions.^29^ Researchers have been captivated by the elegance of their thermal polymerization and the intricate dynamics that arise from it for more than 50 years.^30^ Continued research continues to uncover the lifelike capabilities and practical applications of proteinoids in the field of synthetic biology^31^ and unconventional computing.^32^

The proteinoids’ synthesis in abiotic conditions, biomimetic characteristics, and complex assembly give hope that scientists can understand the progression from inanimate chemistry to evolving biology.^33^ The synthetic polymers provide insights into the long path from primordial chaos to biological organization.^34^

The scanning electron micrographs displayed in Figure 1 provide a visual representation of the various morphological features of synthetic proteinoids. Scanning electron microscopy (SEM) specifications are provided for each image (A–D). High Voltage (HV) indicates the accelerating voltage of the electron beam, with 2.00 kV used for all images to optimize surface detail. The magnification (mag) ranges from 30,000× (image A) to 300,000× (image C), allowing visualization of structures at various scales. Spot size (spot) refers to the electron beam diameter, affecting resolution and scan time. Images A, B, and C have a spot size of 2.0, while image D has a spot size of 2.5. Scale bars provide measurement references, with 417 nm for images A, B, and D, and 100 nm for the highest magnification image C. The SEM images were processed using a custom MATLAB algorithm. This algorithm performs grayscale conversion, contrast enhancement, adaptive thresholding for segmentation, and skeletonization to extract and highlight the structural features of the proteinoid spheres. The resulting skeletons are overlaid on the original image in red to emphasize the complex network of interconnections and boundaries within the proteinoid structures. The images depict proteinoid particles undergoing spontaneous self-organization, resulting in a diverse range of biomimetic shapes and systems, spanning from the microscale to nanoscale constructs. Of particular interest is the lifelike characteristics on display across sizes—from near-cell-like spherical twins to dense nanosphere colonies coating microsurfaces reminiscent of membranous tissue to fragmented nanostructures resembling prototypes of intracellular networks. When combined with computer analysis (as indicated in red), the intricate self-assembly process inherent in these proteinoids starts to resemble the microarchitecture observed in living cells. The concept proposed by Fox and Dose^16,30^ of pseudo-metabolizing protocells arising from abiotic precursor chemistry inevitably brings to mind a speculative vision. Despite being artificially created, these proteinoids demonstrate a strong tendency toward hierarchical arrangement and spatial programming, resembling pattern formation processes and principles of biological systems. Additional investigation into their cross-linking interactions may reveal fundamental design patterns that can be applied to the development of adaptable biomaterials and bioelectronic systems.

**Figure 1:**
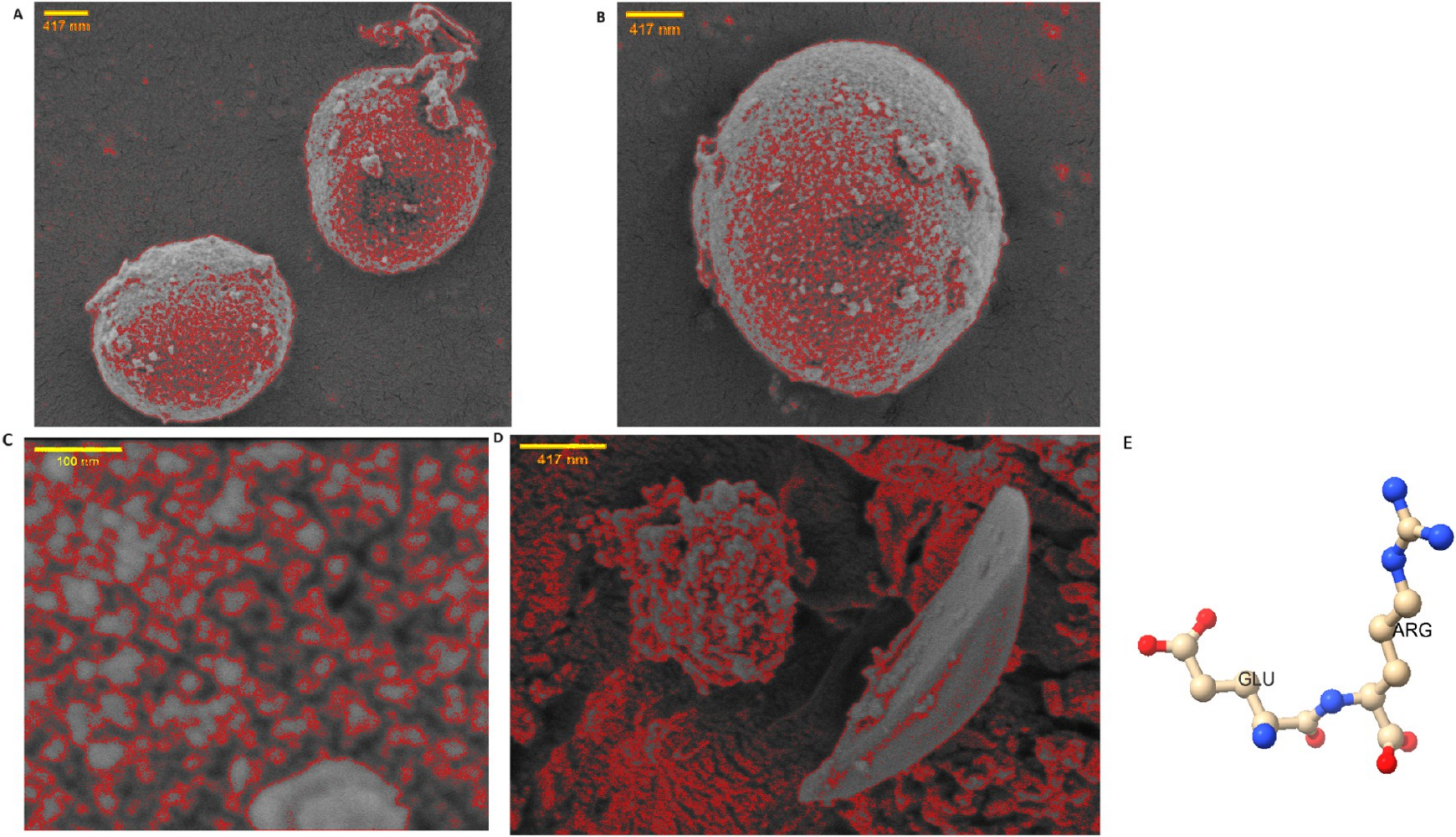
Scanning electron micrographs show various physical characteristics of proteinoid spheres formed through self-assembly. (A) Image depicts two spherical structures in close proximity, each with a diameter of approximately 1.8 μm. (B) A single proteinoid sphere with a width of 2.4 μm is shown, illustrating the formation of microscale structures. (C) Higher magnification reveals densely packed smaller spheres with diameters of approximately 23.5 nm on the surface of a larger microsphere, demonstrating multiscale structural features. (D) The image shows nonspherical particle aggregations, indicating partial breakage of the spherically shaped proteinoid assemblies. This may be attributed to particle stability and degradation processes. These micrographs present different morphologies of synthetic proteinoids, ranging from micron-sized spheres to nanoscale structures. Further investigation of the factors influencing proteinoid self-assembly may provide insights for the design of complex biomaterials. Computational analysis techniques such as segmentation, thresholding, and skeletonization, facilitated by MATLAB, were used to analyze proteinoid boundaries and structures within these samples. (E) Image E shows the chemical structure of a dipeptide l-glutamic acid and l-arginine (l-GLU/l-ARG), which is a crucial component of heat proteinoids.

Additional analysis using high-resolution structural techniques like digital optical microscopy could provide specific properties about the dynamic assembly process, which will assist in the development of bioinspired engineering projects. Proteinoids consistently show similarities between intentionally created artificial systems and the development of organization in early prebiotic chemistry.

### Electrical Properties of Kombucha and Proteinoids

Both Kombucha and proteinoids exhibit patterns of electrical activity similar to that of living neurons.

In ref 35 we demonstrated that Kombucha mats produce action potential-like spikes of electrical potential, the spikes are often grouped in the trains of spikes.^35^ We demonstrated that electrical responses of Kombucha mats to chemical, electrical, and optical stimulation are distinctive and therefore the mats can be used as sensors, or even unconventional computing devices.^35^

Proteinoids show a wide spectrum of oscillations of electrical potential.^21^ The electrical spiking activity of the proteinoids can be modulated with light^31^ and used to implement Boolean gates^36^ and recognition of sounds.^37^

### Tailoring Kombucha with Proteinoids

Materials with sensing and information processing capabilities can incorporate a substrate and a series of functional layers. The functional layers include the active elements where the main electrical activity takes place while the substrate is a solid substance where the functional layers are deposited.^38,39^ Due to low production costs, easy accessibility, flexibility and shape conformability, customization across various scales, renewability and degradability potentials, Kombucha cellulosic films present an excellent substrate candidate for functionalization. On the other hand, proteinoids present unique electrical capabilities such as memristive, memcapacitive, and conductive properties^24^ and are therefore suitable for utilization as functional layers.

Memfractance is a term used to describe the distinct set of characteristics exhibited by memristors, mem-capacitors, and mem-inductors.^37^ Memristors belong to a family of nonlinear dynamical memory devices that can be employed in many applications. They can be used for logic operations and the execution of material implication (IMP), a fundamental Boolean logic operation based on two variables.^40^ These applications encompass the development of logical circuits, stateful logic operations, passive crossbar arrays of memristors for logic operations, memory-aided logic circuits, self-programmable logic circuits, and memory devices. For example, the investigation of mem-fractive characteristics in fungi arises from the possible advantages it presents.^41^ If the strands of fungal mycelium present in mycelium bound composites, along with the fruit bodies, have mem-fractive characteristics, it creates opportunities to include different memory and computer devices directly into architectural construction materials made from fungal substrates. In addition, the idea of wearable fungi that may be used as clothing is still in its initial phases but has demonstrated favorable characteristics including a sleek design, great flexibility, and minimal energy usage in comparison to traditional artificial wearable sensory devices.^42^ Mycelium-bound composites, consisting of organic substrates that have been colonized by fungi, are very promising biomaterials with significant environmental sustainability. These materials are currently being used in applications such as providing acoustic and thermal insulation for wall cladding and packaging.^43^

Memristors are generally considered passive components whose specific functionality is the memristive effect, a history-dependent relationship between charge and flux.^44^ Practical memristor technologies, such as resistive random access memory (ReRAM), utilize nanoscale switches that can modify electrical resistance in a persistent manner.^45^ Memristor components, when included into circuits, facilitate memory, learning, and self–adaptation capabilities. Memristors possess distinctive attributes that offer potential for groundbreaking utilization in memory storage, neuromorphic computing, and other domains.^46^

Although conceived of decades ago, the experimental realization of optimal memristors has been a formidable obstacle. HP Laboratories documented the observation of memristive phenomena in titanium dioxide thin coatings in 2008.^47^ The publication of this correlation between ReRAM devices and Chua’s memristor suggested that memristors have the potential to revolutionize the field of electronics.^48^

However, some researchers contend that it may be physically impossible to create ideal memristors.^49^ Chua expanded the definition to encompass all nonvolatile memory associated with resistance switching, asserting that memristors represent the most primal component of a circuit. However, there are ongoing skepticisms regarding the existence of reality as opposed to merely mathematical constructs. There have been propositions for experiments to determine whether a genuine memristor can be obtained.^49–53^

In brief, although forward-thinking, the memristor continues to be a subject of debate. Enhancing the memorization capabilities of ReRAM and related technologies holds the potential to realize the theoretical potential of adaptive circuitry. Interdisciplinary cooperation among material scientists, circuit theorists, and device engineers is incentivized to address these fundamental inquiries in order to achieve a more comprehensive understanding of the scientific principles that underpin electronic innovations.

Modulating the electrical properties of Kombucha^42^ with proteinoids can lead to the production of a composite material with synergetic computational functionalities such as signaling dynamics, Boolean logic operations, adaptive learning behaviors and sensory transduction mechanisms. By optimizing parameters such as the composition of the Kombucha substrate and functional proteinoid layers and the concentration of proteinoids in the substrate, the computational functionalities and electroactive activity of the Kombucha and proteinoid composites can be tailored.

The potential computational properties emerging from the Kombucha Proteinoid biofilms are summarized in the mind map shown in Figure 2.^24,42^ As highlighted, the symbiotic living system exhibits capabilities spanning neural-like signaling dynamics, Boolean logic operations, adaptive learning behaviors, sensory transduction mechanisms, and potential for unconventional computing architectures. The mind map conveys relationships between these lifelike information processing properties arising synergistically through the hybrid fusion of proteinoids and electroactive bacterial cellulose.

**Figure 2:**
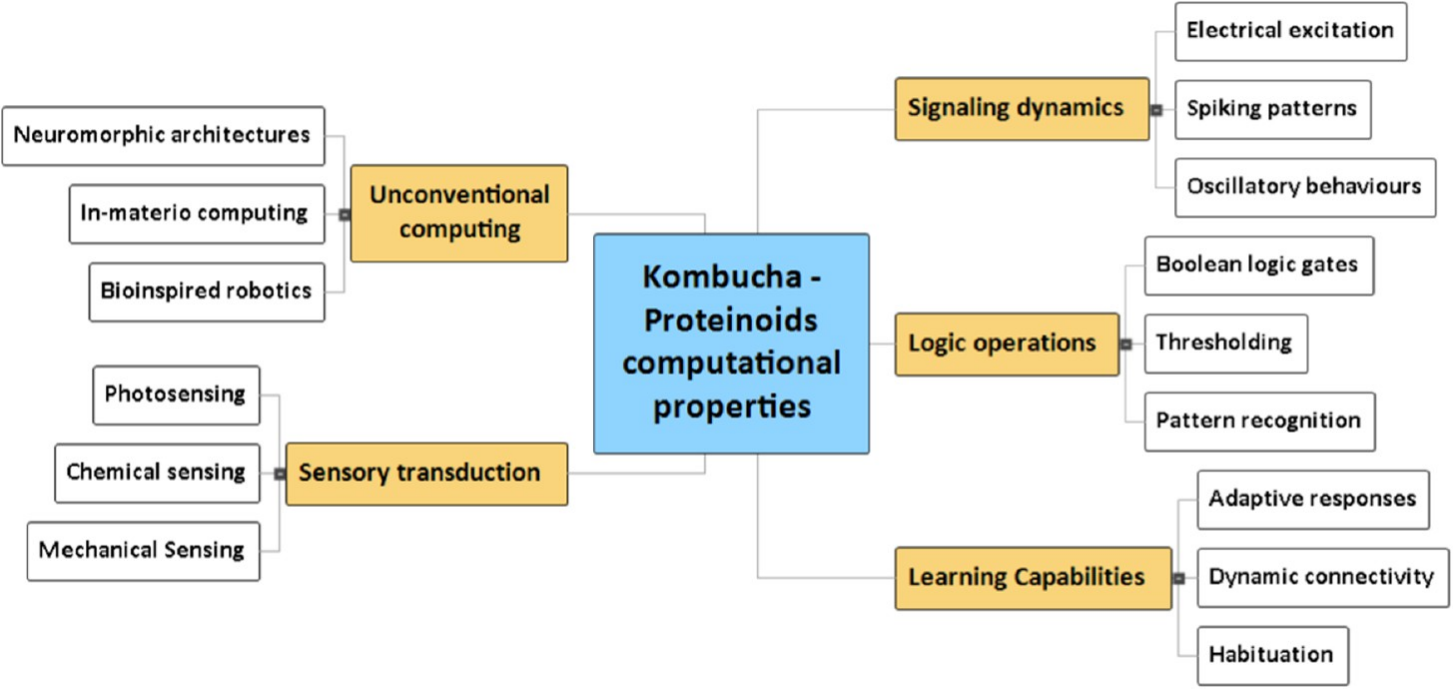
Computational features in Kombucha biofilms depicted in a mind map. The mind map emphasizes the prominent signaling, reasoning, learning, sensory, and unconventional computing characteristics demonstrated by the living electronic composites. The hierarchical structure visually represents the connections between the emergent features of dynamic biomaterials, which are used for information processing and computation. The diverse capabilities emerge from the combination of proteinoids with the bacterial cellulose matrix. Additional clarification of the principles that govern the lifelike bionic behaviors of biofilms holds the potential for ongoing advancements in bioinspired technology.

## Results

### Electrical Characterization of Proteinoid–Microbial Composite Samples

To assess the electroactive properties of Kombucha–proteinoid composites, it is necessary to conduct electrical measurements using techniques such as current–voltage profiling, impedance spectroscopy, and capacitance recordings. The composites are interfaced with platinum–iridium wire electrodes, through which voltage ranges of −1 to +1 V are applied.

High impedance data acquisition systems are widely used in various applications. Picolog loggers and Keithley source meters allow for the analysis of the electrical activity of the films. The recorded current–voltage curves offer valuable insights into conductivity, memristance, and spiking dynamics. The impedance and capacitance profiles provide insights into the response to alternating current and the ability to store charge.

The systematic characterization of electrical properties across various operating conditions uncovers valuable insights into the relationships between stimulus patterns and Kombucha proteinoid network behaviors. Programming desired responses is made possible by adjusting stimulation parameters and material compositions. The input–output mappings in this text highlight the potential for information processing, learning, and logical operations.

Current research is focused on understanding how the nanostructure and integrated physiology of composites contribute to their emergent electronic properties. This knowledge will greatly contribute to the advancement of rational design for customized bioelectronic devices and systems. The combination of microbial matrix, synthetic polymers, and electronics demonstrates exciting potential at the intersection of biology and technology.

Electrical measurements are essential for understanding the intricate mechanisms of Kombucha–proteinoid composite. The understanding of stimulus-response couplings, signal transmission modes, and programmable behaviors in hybrid bioelectronics has greatly advanced both fundamental research and practical applications.

The experimental apparatus used to assess the electrical activity of the composites is shown in Figure 3. The spike patterns in Kombucha films are examined in Figure 3a. The investigation of composite Kombucha–proteinoid biofilms is shown in Figure 3b, with the aim of assessing the impact of the proteinoids that are present. The bioelectrical phenomena observed in our Kombucha-proteinoid circuits were measured using the experimental setup illustrated in Figure 3c. This setup, consisting of adjustable electrodes immersed in the Kombucha-proteinoid culture, connected to a high-precision data logger and controlled by an environmental regulation system, allowed us to detect and analyze microvolt-range signals under various conditions.

**Figure 3:**
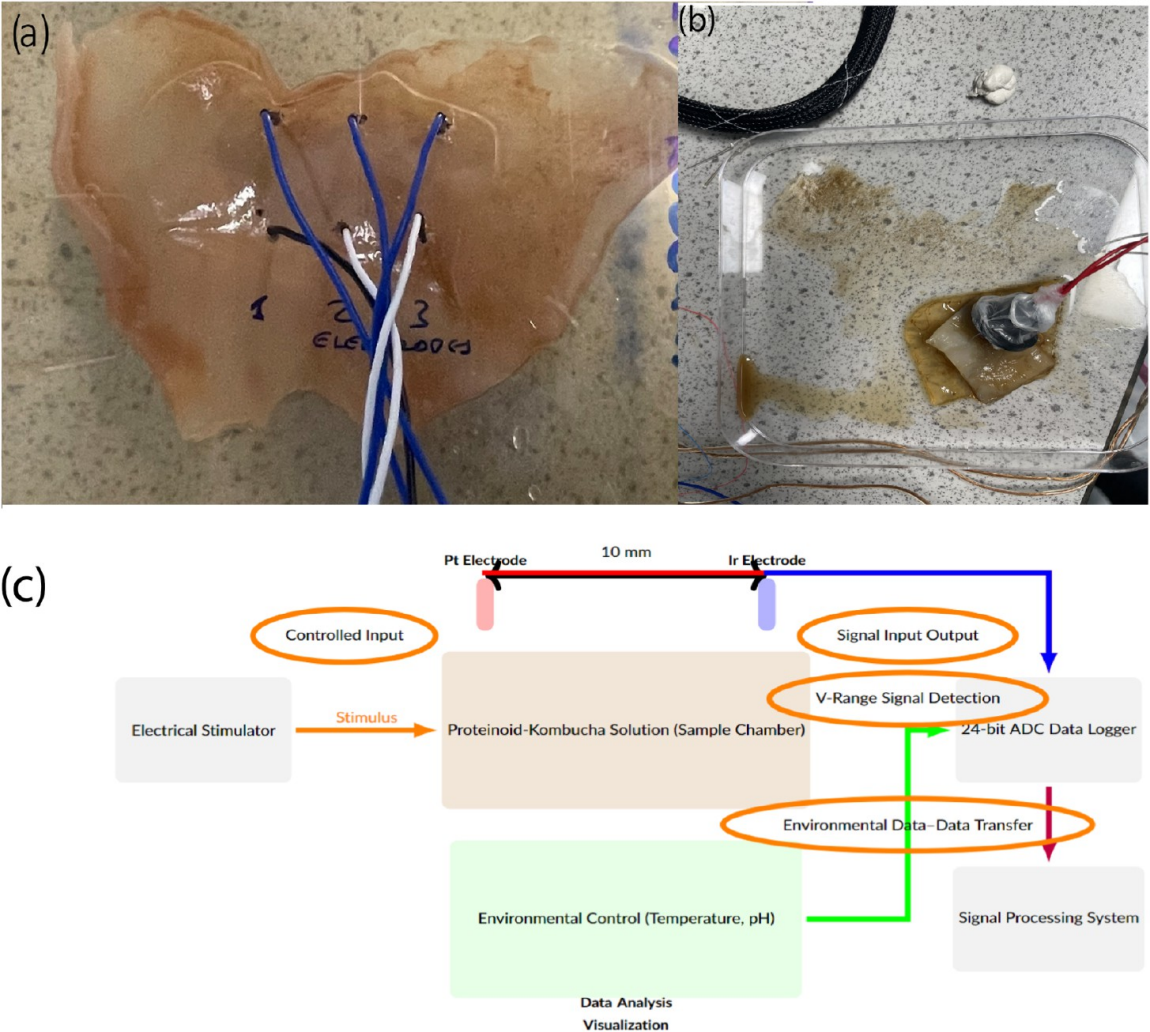
Experimental configurations for examining electrical activity in Kombucha–Proteinoid composite. (a, b) Utilizing platinum–iridium wire electrodes, a measurement configuration is employed to investigate the occurrence of electrical spikes in Kombucha films. (c) Experimental setup for investigating bioelectrical phenomena in Kombucha–Proteinoid circuits. The central container (brown) holds the kombucha-proteinoid culture. Two electrodes (red and blue) are positioned at an adjustable distance within the culture to detect and induce bioelectrical signals. A high-precision data logger records responses in the microvolt range, while an environmental control system (green) regulates parameters such as temperature and pH. Controlled inputs via a separate pair of platinum–iridium wire electrodes is provided to the biological system with the use of an electrical stimulator. The data analysis system processes the collected information, enabling comprehensive analysis of the bioelectrical responses. This setup allows for the study of information processing and communication in kombucha-proteinoid networks under various environmental conditions and stimuli, potentially revealing insights into the behavior of these unique bioelectronic systems.

### Current–Voltage Plots Show Signature of Memfractance

A memristor is an electrical component with two terminals that establishes a relationship between electric charge and magnetic flux connection. This concept was initially introduced by Leon Chua in 1971.^54^ The memristor, along with the resistor, capacitor, and inductor, is one of the essential nonlinear circuit components. Memristance is the measure of electrical resistance in a memristor, which is capable of changing depending on the past passage of current through the device.^55^

The unique electrochemical characteristics resulting from the integration of Kombucha and proteinoids are demonstrated by the analysis of *I*–*V* measurements, as depicted in Figure 4. The current–voltage (*I*–*V*) characteristics of pure l-Glu/l-Arg proteinoids and the Kombucha composite comprising l-Glu/l-Arg are shown in Figure 4a,b. Figure 4c,4d presents a statistical summary of the current distributions for each sample.

**Figure 4:**
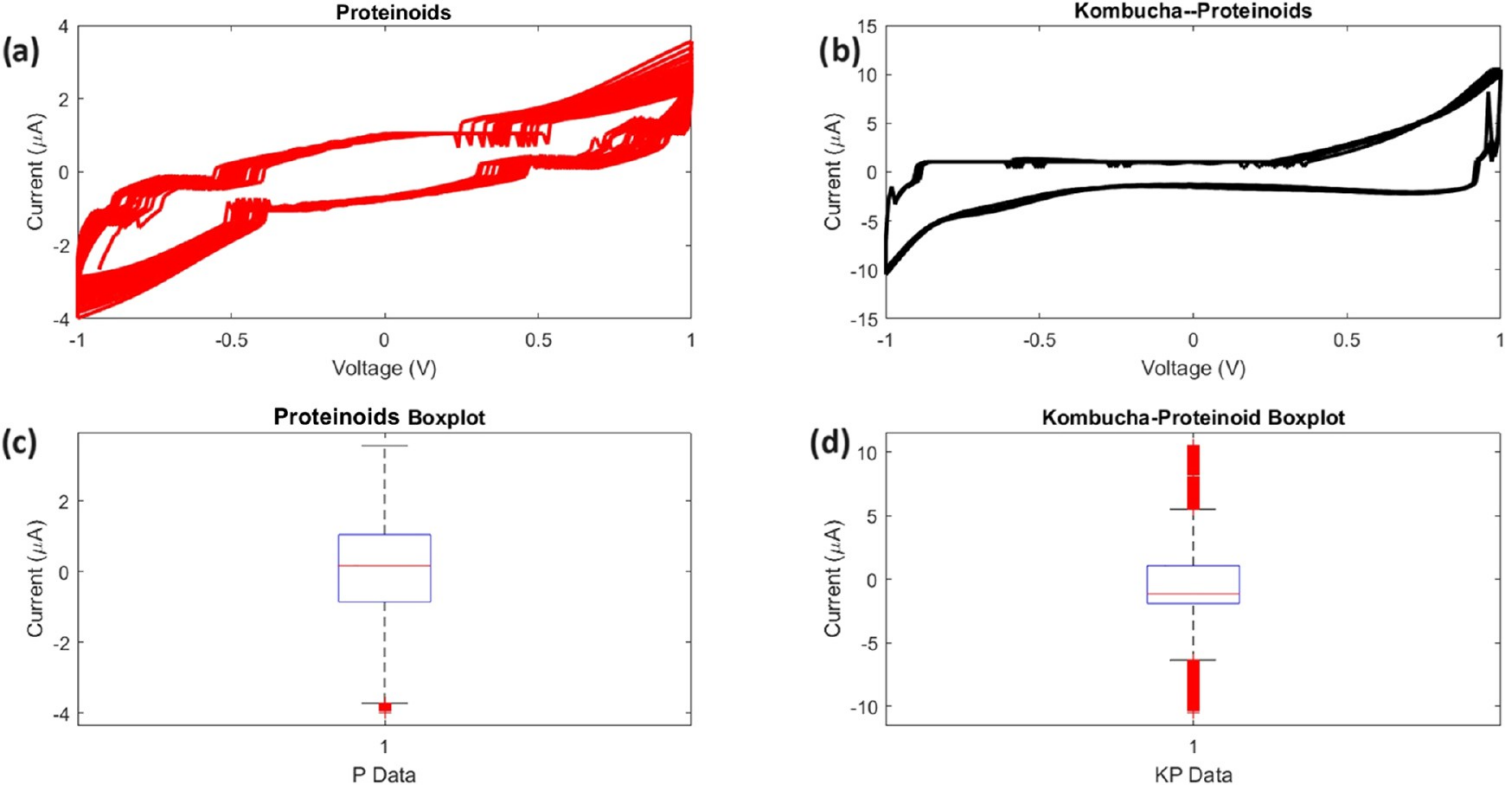
Current–voltage (*I*–*V*) characteristics and statistical analysis of proteinoid and Kombucha–Proteinoid samples using cyclic voltammetry. We performed measurements at room temperature (25 °C) with a scan rate of 50 mV/s, collecting data points at 10 ms intervals. For each sample type, we repeated measurements on *N* = 10 independent samples, with *N* = 20 voltage sweeps per sample. (a) *I*–*V* curve for pure l-glutamic acid/l-arginine (l-Glu/l-Arg) proteinoid sample. The *x*-axis shows applied voltage (*V*) and the *y*-axis shows measured current (μA). (b) *I*–*V* curve for Kombucha microbes combined with l-Glu/l-Arg proteinoids (Kombucha–Proteinoid composite). Axes are the same as in (a). (c) Box plot comparing current distributions of proteinoid (P) and Kombucha–Proteinoid (KP) samples. Each box plot represents the aggregated data from all voltage sweeps across all samples. The box represents the interquartile range, the line inside the box is the median, and whiskers extend to the minimum and maximum values excluding outliers. (d) Statistical metrics for both samples, including mean current, standard deviation, skewness, and kurtosis. The proteinoid sample (P) showed a lower average current (−0.03 μA) compared to the Kombucha–Proteinoid sample (KP) (−0.15 μA). KP exhibited higher standard deviation (3.25) than P (1.40), indicating greater current variability. The P sample showed slight negative skewness (−0.39), while the KP sample had positive skewness (0.40). Kurtosis values were 2.80 for P and 4.53 for KP, suggesting different distribution shapes. These differences highlight the impact of combining synthetic proteinoids with microbial life on electrical behaviors and nonlinear interactions.

The Kombucha composite has a lower average current, but its standard deviation (3.25 μA) is more than double that of the pure proteinoid sample 1.40 (μA). This suggests that the microbial–synthetic composite has a wider and more varied distribution and range of activity, which can be attributed to the combined and mutually enhancing actions of its two components. The enhanced bioelectronic behaviors highlight the advantages of combining biological and synthetic systems to achieve new and advanced functionalities.

In summary, Figure 4 shows the *I*–*V* characteristics and their statistical analysis. Collecting the data requires careful attention to voltage-dependent sampling effects. The current measurements *I*_*i*_ at each voltage point *V*_*i*_ were collected across multiple voltage sweeps. Higher data density occurred near vertical segments of the *I*–*V* curves due to cyclic voltammetry. The box plots give a general view of current distributions. But they should be used with caution. They aggregate measurements from different voltage regions with different sampling densities. Nevertheless, the data reveals notable statistical disparities in the *I*–*V* characteristics resulting from the incorporation of proteinoids into the microbial Kombucha matrix. This showcases adjustable electronic characteristics and enhanced functionalities compared to the separate components.

The analysis of higher order statistics provides valuable insights into the shape and symmetry of current distributions observed in the l-Glu/l-Arg proteinoid and Kombucha–Proteinoid composite samples. The pure proteinoid sample exhibits a kurtosis value of 2.798, suggesting a distribution that is flatter with wider peaks in comparison to a normal distribution. The data also indicates a negative skewness of −0.39, indicating a slight left-skewed asymmetry.

On the other hand, the Kombucha composite shows a higher kurtosis of 4.53, which is much more similar to a Gaussian distribution. The skewness value of 0.40 indicates a relatively balanced current density distribution. The data suggests that the combination of Kombucha and proteinoid leads to a distribution that is more tightly concentrated around the mean, with less pronounced tails.

### Conductivity Changes with Frequency

Understanding the electrical characteristics of Kombucha–Proteinoid composite biofilms necessitates the use of precise methods for characterizing charge transport. Conductivity measurements conducted on disordered organic systems are susceptible to experimental artifacts that have the potential to render the underlying models incorrect. For instance, the electrical characteristics of melanin were formerly believed to be due to its amorphous semiconductivity. However, further analysis has shown problems with the models that assume hydrated dielectric qualities.^56^

To prevent these errors, it is necessary to apply strictness in aspects such as electrode arrangements, sample shape, biassing conditions, and equilibrium dynamics. An in-depth analysis of the relationship between frequency and field strength in conduction across various time intervals provides a better understanding of transport mechanisms. It is crucial to take into account charge injection, trapping, recombination, and interfacial effects.

The presence of multiscale structure at the molecular, nano, micro, and macro levels adds complexity to the understanding of Kombucha-proteinoid conductivity. The diverse, structured, and water-rich biofilm morphology impacts the flow of electrical current and the spread of its effects. By combining spectroscopy, microscopy, and calculations with conduction testing, it becomes possible to establish a correlation between the structure and function.

The frequency–dependent conductivity profiles of the proto–brain samples provide insights into their tunable electrical behaviors. The conductivity σ was derived from the measured capacitance values using the following relation

1 $$\sigma =\frac{1}{\omega C\sqrt{1+\tan^{2}\left(\phi \right)}}$$

Where ω is the angular frequency, *C* is the capacitance, and ϕ is the phase angle between current and voltage. This accounts for both resistive and reactive contributions over the test frequency range of 0 to 300 kHz.^22^ The conductivity equation comes from an equivalent circuit model. It is a parallel *RC* circuit. Here, *R* is the biofilm’s resistance and *C* is its capacitance. The impedance *Z* of this parallel RC circuit is given by

2 $$Z=\frac{R}{1+j\omega \mathrm{RC}}$$

From this, we can express the conductivity σ in terms of the measured capacitance *C* and phase angle ϕ (where tan­(ϕ) = ω*RC*). This gives eq 1. This model assumes: (i) uniform material properties, (ii) ideal capacitors and resistors, and (iii) negligible inductance effects at the measured frequencies.

The conductivity measurements of mixtures including Kombucha and proteinoids exhibited behaviors that were dependent on the composition and selective with respect to frequency. The Kombucha–Proteinoid (KP) sample had the highest conductivity across frequencies, suggesting a higher degree of charge mobility. Typically, the conductivity decreased as the frequency increased due to hindered charge transport. Nevertheless, there were noticeable surges in conductivity seen for the Kombucha and Kombucha–Proteinoid films throughout the frequency range of 150–250 kHz, exhibiting a remarkable rise of up to 1000 times from 0.03 to 22 S/cm. This suggests the initiation of heightened conduction processes at particular resonance frequencies. The proteinoid sample, in its pure form, demonstrated a distinct and rapid rise in conductivity between the frequencies of 152–259 kHz. The results emphasize the adjustable electrical conduction properties of the composite biofilms, which may be customized by manipulating input signal characteristics such as frequency. The conductivity spectra demonstrate the ability to choose control frequencies by assembling microbial and synthetic components in a modular manner. Additional clarification of the conduction mechanisms will assist in the logical design of bioelectronic devices with intentional, responsive functionality. The composition-dependent, frequency-selective conduction properties of the Kombucha–proteinoid biofilms are highlighted in Figure 5.

**Figure 5:**
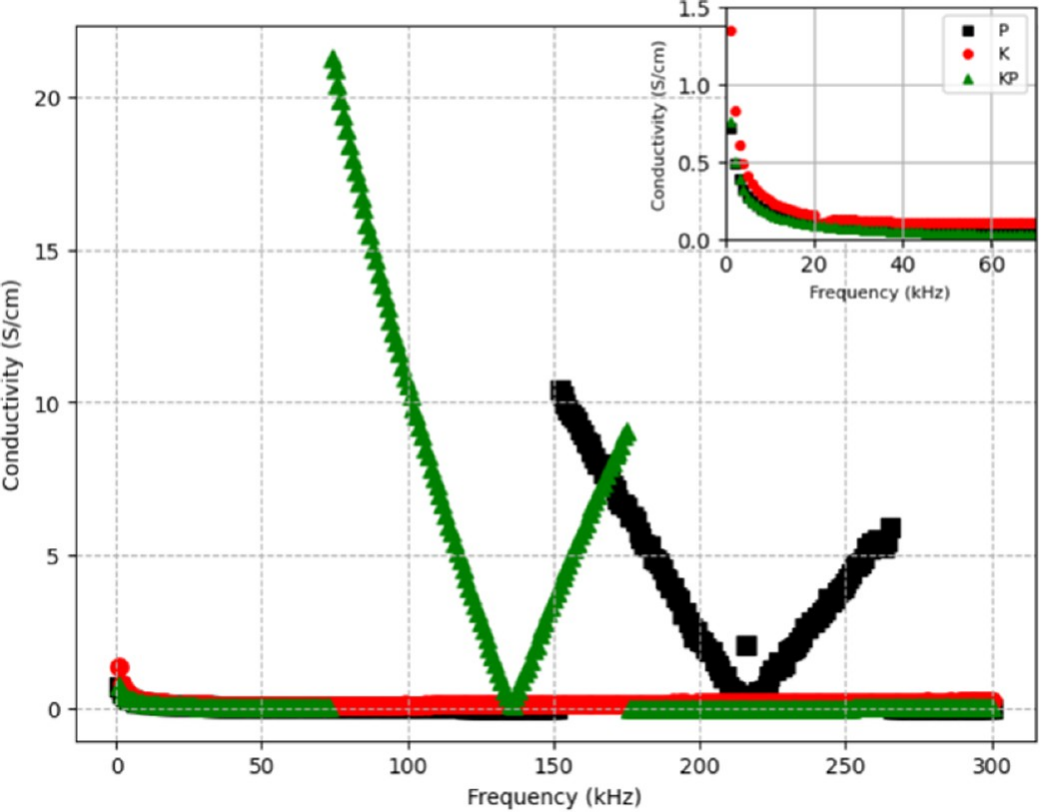
Frequency-dependent conductivity of Kombucha–proteinoid composite biofilms. Conductivity spectra are shown for samples with compositions including Kombucha (K), Kombucha–Proteinoid (KP), and proteinoid (P). The KP and P sample exhibits the highest conductivity over the frequency range. Sharp increases in conductivity occur for the KP films between 74–178 kHz, rising over 1000× from 0.03 to 20 S/cm. A comparable spike occurs for sample P from 152–259 kHz. The tunable conductivity dynamics demonstrate activation of selective charge transport mechanisms at specific resonant frequencies. The excitable dynamics are evidenced by several key features: (1) the presence of sharp conductivity peaks indicating threshold-like responses, (2) the frequency-dependent activation shown by distinct spikes at specific frequencies (74–178 kHz), and (3) the return to baseline conductivity after each excitation event—a hallmark of excitable systems. These response traits match classical excitable system behavior. Subthreshold inputs cause minimal responses. Superthreshold stimuli trigger full activation. All measurements were performed using a BK Precision 891 LCR meter with maximum frequency capability of 300 kHz, set to autorange, at a test frequency of 1.000 kHz, with a signal level of 1 VRMS (root-mean-square voltage) and slow measurement speed to ensure accuracy.

Figure 6 shows a statistical analysis of conductivity measurements. It compares three sample types: proteinoids (P), Kombucha (K), and Kombucha–proteinoid composites (KP). Panel A shows normal distribution fits for conductivity measurements (*n* = 300 per sample type). The inset table has the mean (μ) and standard deviation (σ) values. Conductivity measurements were taken at 1 Hz intervals under constant temperature (25 °C) and humidity (60%). Panel B shows box plots of the same data. The boxes are the interquartile range (IQR). The horizontal line is the median. The whiskers extend to 1.5 × IQR. Red crosses mark the outliers. Each box plot summarizes measurements from *N* = 10 independent samples, with *n* = 30 measurements per sample.

**Figure 6:**
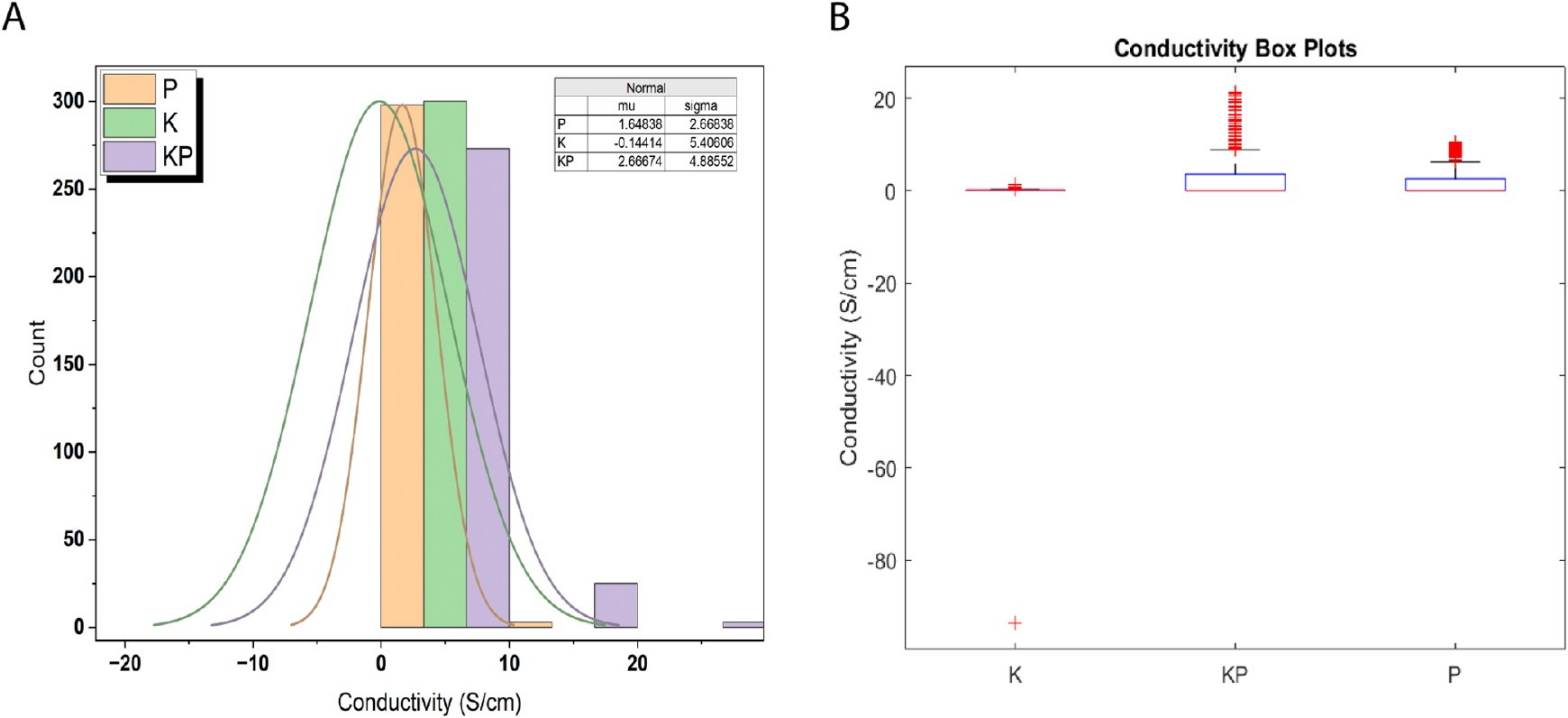
(A) Fitting normal distributions allows quantification of central tendency and variability within the measured conductive responses across proteinoid (P), Kombucha (K), and composite (KP) samples. The proteinoid-only sample possesses a baseline mean conductivity of 1.65 S/cm with a standard deviation of 2.67 S/cm characterizing its distribution spread. By contrast, the mixed Kombucha–Proteinnoid sample has an elevated mean of 2.67 S/cm and increased variance with a standard deviation of 4.89 S/cm. This divergence demonstrates that interfacing the synthetic protocells with the living microbial biofilm significantly amplifies the diversity and range of resultant bioelectronic behavior beyond either standalone system. However, the Kombucha sample exhibits an outlier distribution with comparable variance (σ = 5.41 S/cm) yet inverted mean conductivity centered around −0.14 S/cm constituting a flipped response relative to the prototypical proteinoid profile. (B) Quantiles of conductivity for compositions of Kombucha and proteinoids. The boxplots display the distribution of conductivity (S/cm) values for samples K, KP, and P. The 25th, 50th, and 75th percentile values are plotted, indicating that KP has the largest range, reaching approximately 20 S/cm, while K, and P are distributed below 2.62 S/cm. The extensive distribution ranges emphasize the adjustable conduction characteristics of KP biofilms resulting from the selective incorporation of both microbial and synthetic elements. Conductivity distributions can be statistically profiled to identify specific design approaches for customizing bioelectronic response behaviors.

Probability distributions characterize the relative likelihood of variable values (Figure 6A). Gaussian models offer useful reference predictions given by

3 $$f\left(x|\mu ,\sigma_{N}\right)=\frac{1}{\sigma_{N}\sqrt{2\pi }}e^{-\left({\left(x-\mu \right)}^{2}/2\sigma_{N}^{2}\right)}$$

where μ and σ_*N*_ denote the mean and standard deviation, respectively. When comparing fitted normal distributions, differences in central tendency and dispersion properties emerge. The mean conductivity of the proteinoid-only sample (P) is 1.65 S/cm, with a standard deviation of 2.67 S/cm. In comparison, the Kombucha–proteinoid composite (KP) has a greater mean of 2.67 S/cm and a bigger variance with σ = 4.89 S/cm. This suggests that combining proteinoids with the microbial Kombucha matrix greatly increases the diversity and extent of conductive responses. The Kombucha sample (K), on the other hand, has comparable variance but an inverted mean conductivity centered about −0.14 S/cm, indicating an outlier distribution. The goodness of fit test would determine whether Gaussian models accurately described the sample data. Further statistical analysis could evaluate the relevance of differences in conductivity distribution parameters among samples. Using probability density profiles to characterize emergent behaviors demonstrates how coupling synthetic biology with living microbial scaffolds alters the final bioelectronic phenotypes.

The research demonstrates a notable increase in conductivity achieved with the incorporation of proteinoids into the Kombucha matrices. The significant improvement in conductivity by selective hybridization (Figure 6B) prompts the need for further optimization in order to cater to specific bioelectronic applications. The distributions measure ways to modify emergent response behaviors by adjusting Kombucha–proteinoid ratios.

### Controlling Material Functionality

The Kombucha–Proteinoid composite films offer exciting possibilities for the development of low power analogue integrated systems, drawing inspiration from biology. These soft living electronic materials offer the unique benefits of biological intelligence, such as adaptation, autonomy, and fault tolerance, instead of relying on traditional rigid electronic components.^51^ The integration of synthetic biology and semiconductor electronics opens up exciting possibilities for the development of advanced chip technologies.

The Kombucha–Proteinoid composites have the remarkable capability to generate electrically excitable signals and adapt their conduction properties. Figure 5 clearly shows that the Kombucha-proteinoid composites display electrically excitable responses that are adjusted by stimulus frequency. The conductivity of the KP biofilms increases over 1000× when the input frequency is varied from 74 to 178 kHz, as seen by the spectra. This selective spiking behavior suggests a possible mechanism involving ionic transport pathways within these mixed microbial–abiotic networks. Further studies are needed to confirm how conductivity is modulated. Similarly, sweeping the input frequency from 152 to 259 kHz causes a sharp conductivity amplification driven by frequency-dependent charge conduction pathways inherent in the proteinoids’ assembly architecture. These tunable nonlinear voltage enhancements confirm Kombucha–Proteinoid systems’ potential to electrically excite internal conductive regions in ways that increase output responsiveness. The emergent features go beyond the individual ingredients, with the interaction of the microbial cellulose matrix with synthetic protocell microspheres yielding complicated excitation dynamics analogous to excitable cells. In the same manner that neurons exhibit selective firing thresholds controlled by input stimulation patterns, mixed abiotic–biological composite networks self-organize complementary pathways that transform and relay external signals across a wide range of spatiotemporal regimes.

These networks can be used to create flexible analogue circuits. Characteristics like photosensitivity in circuits allow for the adjustment of their behavior and performance based on inputs, much like the processes of natural synaptic plasticity. This provides a way to create hardware systems that closely mimic real–life systems and possess the capability to learn and optimize themselves.

Beyond components, Kombucha–Proteinoid biofilms offer models to inspire wholly new bio–inspired device concepts. Studying the emergent complexity of microbial–abiotic symbiosis and specialized signaling cell types reveals design principles alternative to individual transistors.

The response dynamics of Kombucha–Proteinoid biofilms can be tuned by their compositional ratios, as evidenced by characterization of input and output signals for films with varying proteinoid and Kombucha content.

In summary, Figure 7 highlights how modulating proteinoid–microbial ratios predicts tunable input–output transformations, enabling custom tailoring of response dynamics in these composite living materials. Figure 7 depicts the electrical responses of two distinct samples. Figure 7A depicts the first sample, which contained 40% proteinoids and 60% Kombucha. Figure 7B depicts the second sample, which contained 25% proteinoids and 75% Kombucha. The amplitude and frequency of the input and output signals were both measured. The input for the 40:60 ratio sample had an amplitude of around 5082.71 mV and a frequency of 0.02 Hz. The output signal, on the other hand, was significantly weaker, with an amplitude of only 229.08 mV and a frequency of 0.02 Hz. The input in the 25:75 ratio sample had a similar amplitude of roughly 5094.31 mV and a frequency of 0.02 Hz. The output of this sample had an amplitude of 219.60 mV and a frequency of 0.02 Hz. Comparing the two samples reveals that the one containing more proteinoids (40:60) produced a slightly higher output amplitude, while both maintained the same frequency. This shows that altering the mixture ratio has an effect on the proteinoid–Kombucha system’s computational capability.

**Figure 7:**
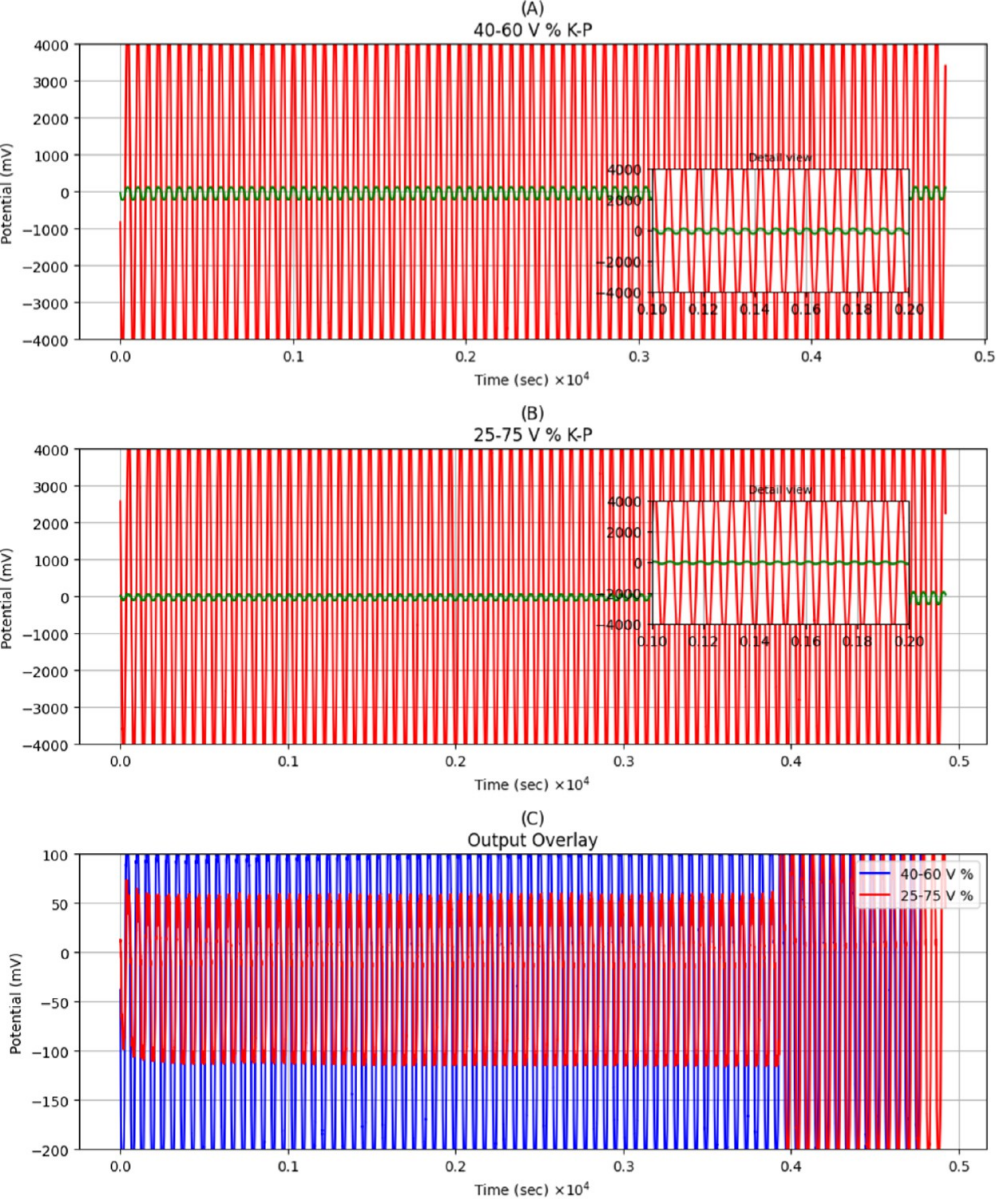
Input is a sinusoidal signal, and the output is the response of the Kombucha-proteinoids to this input. The plots depict the input and output signals for Kombucha–Proteinoid biofilms with different composition: (A) 40% proteinoids and 60% Kombucha (KP), (B) 25% proteinoids and 75% Kombucha. The insets show detailed views of a single input-output cycle, highlighting the phase relationship between input (red) and output (green) signals and revealing the characteristic response pattern of each composition. The key input and output parameters derived from the signals encompass amplitude and frequency. The 40:60% KP sample had an input amplitude of 5082.71 mV and a frequency of 0.02 Hz. The output had a decreased amplitude of 229.08 mV, although a frequency of 0.02 Hz. The 25:75% KP sample had an input with an amplitude of 5094.31 mV and a frequency of 0.02 Hz. The output had a decreased amplitude of 219.60 mV and a frequency of 0.02 Hz. The different response amplitude between the 40:60% and 25:75% KP samples indicates that the ratio of proteinoid–Kombucha (KP) composition affects the computational features of the system. (C) The output potential signals (mV) were recorded over time with Kombucha ratios of 40:60 and 25:75 by volume.

We analyzed the voltage time series using Shannon entropy. It measures the unpredictability of signal variations. The voltage amplitudes were split into 8 bins. Then, we calculated the entropy

4 $$H=-\sum p_{i}\log_{2}\left(p_{i}\right)$$

where *p*_*i*_ is the probability of voltage values in bin *i*. The 25–75% (v/v) KP mixture displayed a higher level of randomness in its voltage compared to the 40–60% (v/v) KP sample, with a value of 1.02 bits vs 0 bits. However, while conducting cross-correlations, it was found that there was minimal similarity between the two signal traces. By utilizing percentile cutoffs, the comparison of extremes highlighted the range of outputs between voltage spikes and troughs, encompassing a greater breadth from the 25th to the 75th percentile. By mapping the output paths across phase space, it was revealed that the 40–60 system exhibits more regular cycles compared to the extended and swirling chaos observed in its 25–75 “cousin”. Collectively, our series of testing reveals that recipes with lower proteinoid content and improved Kombucha produce electrical fluctuations that are more chaotic and less predictable. In simpler terms, increasing the presence of microorganisms and reducing the amount of synthetic components seems to enhance the complexity of this mixture. The adjustment of biological and non–biological elements provides a means to potentially fine–tune the development of advanced cognitive abilities through the construction of next–generation biological systems.

### Building Logic with Kombucha–Proteinoid Proto Brain

Recent research has shown that Kombucha-proteinoid composite biofilms have the ability to carry out basic Boolean logic operations, including as AND, OR, XOR, XNOR, NAND, and NOR gates.^35,57^ Converting the continuous electrical outputs into binary signals allows for performing bitwise operations using the basic logic operators. The integration of different network output signals enables the implementation of diverse gates, establishing a fundamental basis for biologic computation.^42,43^

The logic functionality is derived from the intricate spatiotemporal signaling dynamics of the films.^58^ Inputs, such as stimuli or proteinoid doping, undergo a transformation process that results in the formation of interaction patterns. These patterns then perform transfer functions that are similar to Boolean logic. Thresholding is a process that turns continuous outputs into binary levels, which are essential for performing bitwise logic operations. This establishes the following equations that present the mathematical logic formulas. The AND logic operation

5 $$y=x_{1}\wedge x_{2}$$

The OR logic operation

6 $$y=x_{1}\vee x_{3}$$

The XOR (exclusive OR) logic operation

7 $$y=x_{1}⊕x_{3}$$

The XNOR (exclusive NOR) logic operation

8 $$y=x_{1}↔x_{3}$$

The NAND logic operation

9 $$y=\neg \left(x_{2}\wedge x_{4}\right)$$

The NOR logic operation

10 $$y=\neg \left(x_{2}\vee x_{4}\right)$$

where ∧ represents AND, ∨ represents OR, ⊕ represents XOR, ↔ represents XNOR, and ¬ represents NOT. The input variables are denoted as *x*_1_, *x*_2_, etc. and the output is *y*. To convert the continuous output voltage *V*_out_ to binary levels for logic

11 $$V_{out}^{\prime }=\{\begin{array}{l} 1,if\;V_{\mathrm{out}}>0.5\;V\\ 0,if\;V_{\mathrm{out}}\leq 0.5\;V \end{array}$$

where *V*_out_^′^ is the discretized binary voltage used for the logic operations.

Figure 8 illustrates the ability of Kombucha–proteinoid biofilms to conduct basic logical functions. Examples of AND, OR, XOR, XNOR, NAND, and NOR gates are displayed, which are accomplished by bitwise operators and thresholding the biofilm output signals at different input voltages. The strong logic functionality is demonstrated via duplicate gates in various sample output pairs. This basic logic realization highlights the potential of hybrid bio–abiotic materials for information manipulation and biomolecular computing, an important first step toward bio–inspired unconventional computer systems. The goal of ongoing attempts to enhance the logic gates’ generalizability and dependability is to fully use the rich processing capabilities of dynamic biomaterials, which are inspired by biological intelligence. Kombucha–Proteinoid biofilms create analog electrical signals. These signals can be used to make logic gates. In our analysis, we apply thresholding to these analog signals within our MATLAB code to create digital logic outputs. The curves in Figure 8 show the raw electrical responses from the biofilms before thresholding. The MATLAB code uses logical operators like ∧ for AND, ∨ for OR, and ⊕ for XOR. It applies these to the normalized outputs to create the logic gate functionalities. For example, in the AND gates, the code applies the ∧ operator to determine where both inputs would satisfy the logical condition. The XOR implementation (⊕) is particularly interesting as it captures differences between input signals. The raw biofilm responses in the figures might not look like standard truth tables. However, our postprocessing shows that these biohybrid systems can perform several logic functions. These include NAND $\left(\overset{®}{A\vee B}\right)$ , NOR $\left(\overset{®}{A\vee B}\right)$ , and XNOR $\left(\overset{®}{A⊕B}\right)$ . The reproducibility of input pairs (1–2 and 3–4) shows these behaviors are consistent. This holds true even with natural biological differences.

**Figure 8:**
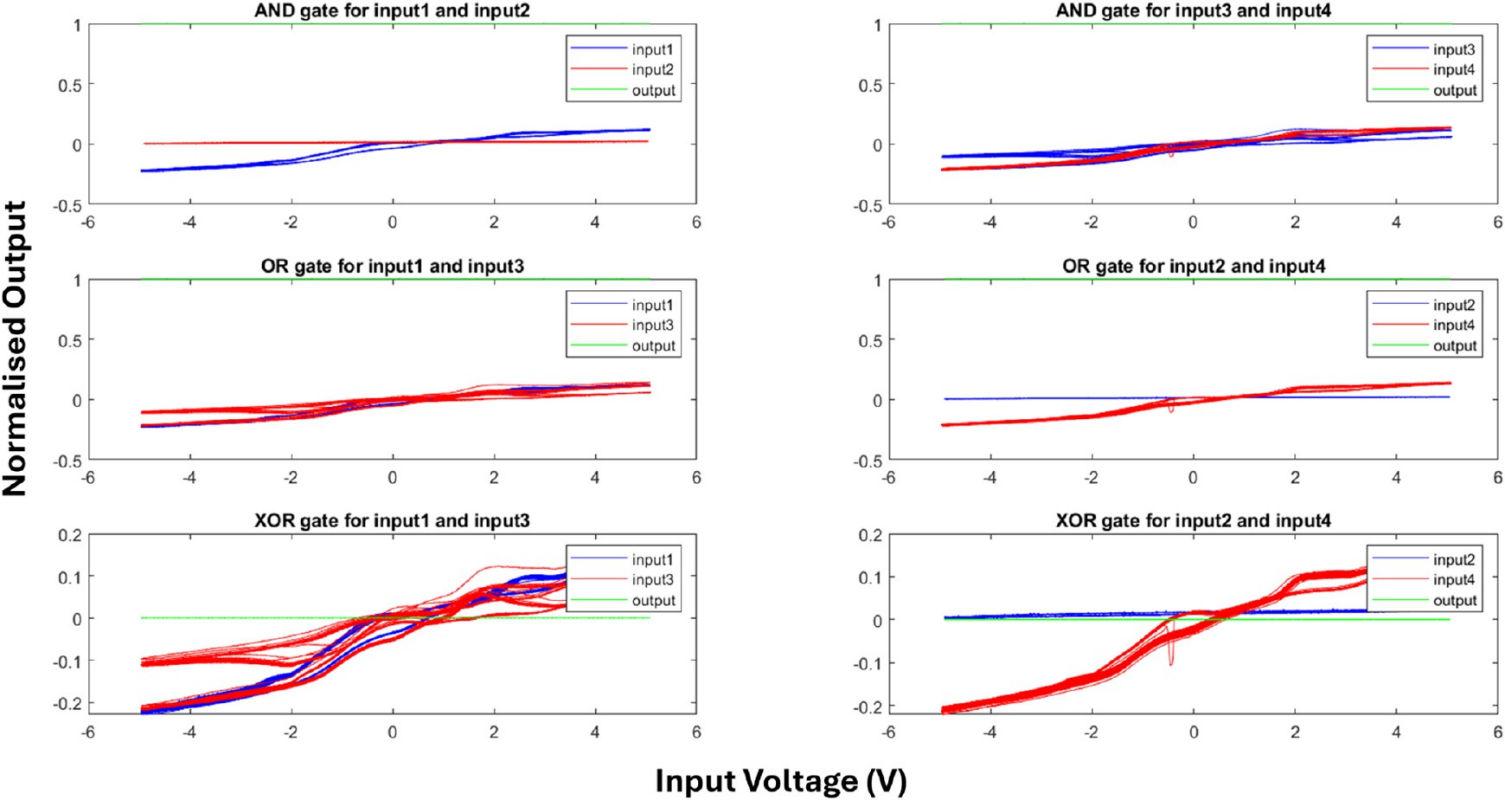
Utilizing Kombucha–Proteinoid biofilms, static logic gates are realized. Each data point represents a steady-state measurement where the *x*-axis shows the applied DC input voltage (−5 to +5 V), and the *y*-axis shows the corresponding normalized output response. The charts demonstrate sample AND, OR, and XOR logic operations carried out by bitwise and thresholding operators on Kombucha-proteinoid output signals. Each curve represents multiple measurements at different fixed input voltages, not time-series data. The *y*-axis represents the normalized output of the biofilms, ranging from 0 to 1. This continuous output is then interpreted as binary states for logic operations, with values above a certain threshold considered as “1” and below as “0”. Logic applied to separate pairs of outputs from biofilm samples is highlighted by duplicate gates.These measurements characterize the steady-state behavior of the biofilms as voltage-controlled logic elements, though future work could explore their dynamic response to time-varying inputs. The ability to modify signals logically highlights the potential of these living hybrid materials for information processing and biomolecular computing. Bio-inspired unconventional computing that uses lifelike dynamics is made possible by the continued development of reliable, generalizable logic gates.

Investigating emergent dynamics in composite biotic–abiotic networks necessitates quantitative mapping of complicated recurring events. Figure 9 shows phase portraits reconstructed from time-delayed coordinates showing complicated attractors driving system dynamics. Phase space reconstruction is a well-established model for understanding processes using geometric coordinates encoded by

12 $$x_{i}={\left[s\left(t\right),s\left(t+\tau \right),\cdot \cdot \cdot ,s\left(t+\left(m-1\right)\tau \right)\right]}^{T}$$

where *s*(*t*) represents the bioelectric signal, τ dictates embedding lag, and *m* is the embedding dimension. Resultant attractor shapes sculpted by recursive patterns reveal multidimensional topology difficult to profile using linear statistics. The 40:60% K–P depiction, for example, reveals a more complex structure, with wider spreads along orthogonal vectors indicating increased stochasticity. Quantitative Lyapunov exponents and entropic measurements support this difference, which is compatible with improved kinetics due to increased microbial input. Fractal Hausdorff estimates, on the other hand, approach an upper complexity bound for both compositions, indicating robust self-organized priority.

**Figure 9:**
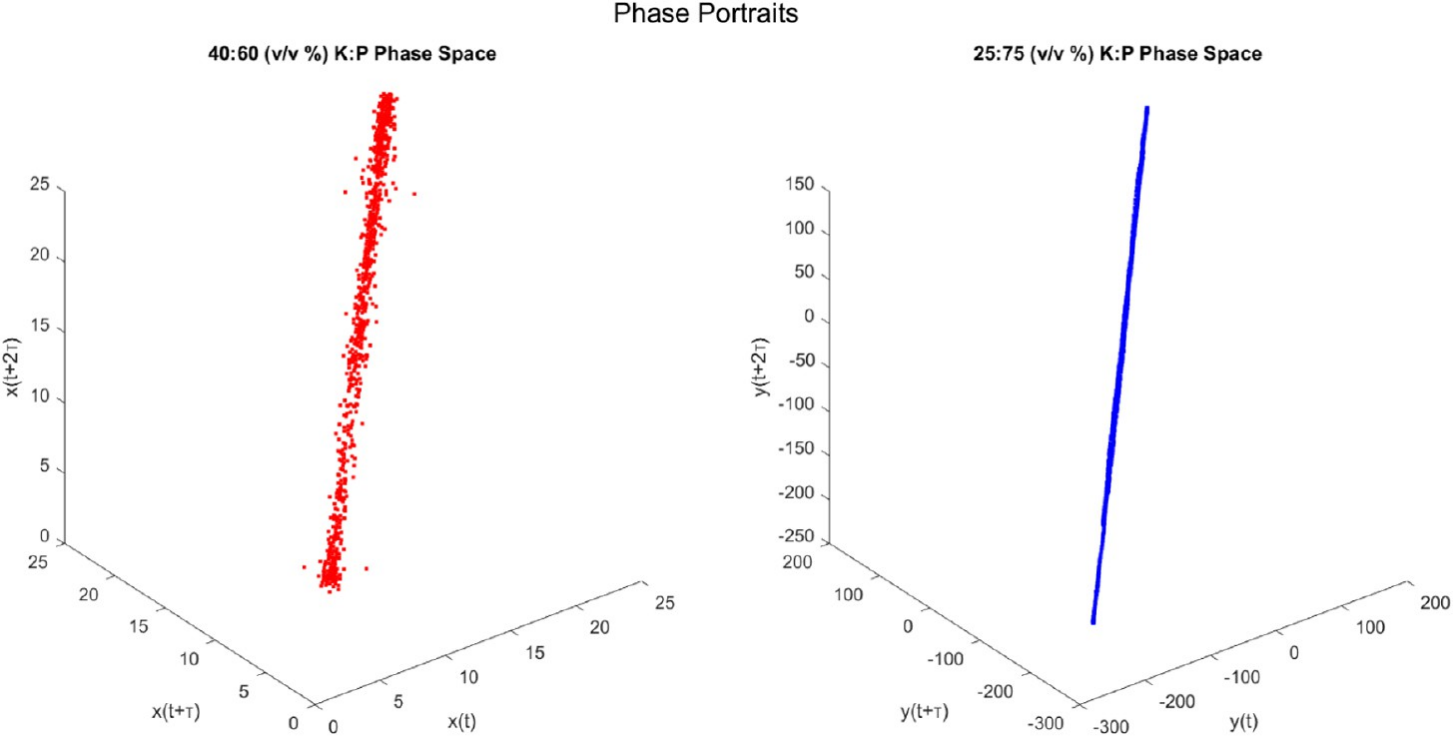
Phase space portraits constructed from single output voltage measurements using time-delay embedding. The measured output voltage *V*(*t*) is plotted against its time-delayed version *V*(*t* + τ), where τ is a fixed delay, creating coordinates *x*(*t*) = *V*(*t*) and *y*(*t*) = *V*(*t* + τ). This technique allows us to reconstruct the dynamical behavior from a single measured variable. The given results are from bio-hybrid composite samples, especially microbial Kombucha matrices merged with synthetic proteinoid microspheres at culture-to-additive ratios of 40:60 and 25:75 (v/v %), respectively. The system was driven by a constant DC bias voltage, and the resulting spontaneous voltage fluctuations were recorded over time. Tracking prior values against current signals reveals geometric attractors, which indicate the processing complexity of each live network. The shape differences between the two ratio scenarios show that the mix of biological and artificial components influences emergent activity profiles. While *x*(*t*) and *y*(*t*) may appear to be different signals, they are actually the same output voltage measurement separated by a time delay τ—a standard technique for visualizing the dynamics of nonlinear systems. Comparing phase pictures of 40:60 and 25:75 allows contrasting the dynamical response diversity for distinct K–P composition balances. Interpreting attractor geometry also helps with tuning.

The largest Lyapunov Exponent (LE) measures the level of unpredictability in dynamical systems, where positive values indicate the presence of chaos. The Kombucha–Proteinoid (K–P) sample with a 40:60%v/v ratio yielded a slightly negative maximal LE of −0.026. The bioelectronic output signal of this suggests a higher degree of order, residing closer to stable fixed point or limit cycle attractor regimes. In contrast, the 25:75% K–P output yielded a significant maximal Lyapunov Exponent of 0.035, indicating the presence of chaotic behaviors and trajectories that are more likely to diverge. The higher concentration of Kombucha enables the emergence of random influences, with small disturbances spreading unpredictably through the conductive composite network.

Entropy is a measure that helps us understand the level of randomness and unpredictability in time series data. The 40:60 K–P sample exhibited a higher entropy value of 0.105, suggesting the presence of more irregular signal patterns. In contrast, the 25:75 K–P system exhibited lower entropy, approximately 0.017, indicating a higher level of repeatability and predictability in its dynamics. Although both compositions exhibited nonlinear bioelectrical behavior, the lower proteinoid fraction led to more orderly and less complex configurations. Adjusting the K–P balance has a significant effect on emergent signaling traits such as regularity and coordination, going beyond basic chaos measures.

Finally, the correlation dimension of Fractal Hausdorff^59^ provides insights into the fractal properties of chaotic signals. Values generally fall within the range of 1 to 2 for fractional nonlinear systems. The correlation dimensions of both the 40:60 and 25:75 K–P samples were approximately 0.995. It suggests that there are certain patterns that exist between regular motion and chaotic trajectory divergence. Although the overall fractality remains intact, the lower proteinoid fraction causes a shift in informative signaling toward disruptive noise.

- How do the initial synaptic weights in temporally coded neural networks, trained on Kombucha–Proteinoid data, shape the overall network architecture and its capacity for unconventional computing?

Figure 10 displays the initial synaptic weights in temporal coding neural networks that were trained using Kombucha–Proteinoid data. The heatmap plots illustrate the synaptic connections between presynaptic and postsynaptic neurons for two distinct compositions: Sample 1 (Kombucha–Proteinoid 40:60% (v/v)) (Figure 10a) and Sample 2 (Kombucha–Proteinoid 25:75% (v/v)) (Figure 10b). The heatmaps utilize colors to reflect the intensity of synaptic connections, with warmer hues denoting higher connectivity. The heatmaps demonstrate unique network structures for each composition scenario, emphasizing the impact of the Kombucha–Proteinoid mixing ratio on the overall arrangement of the neural network.

**Figure 10:**
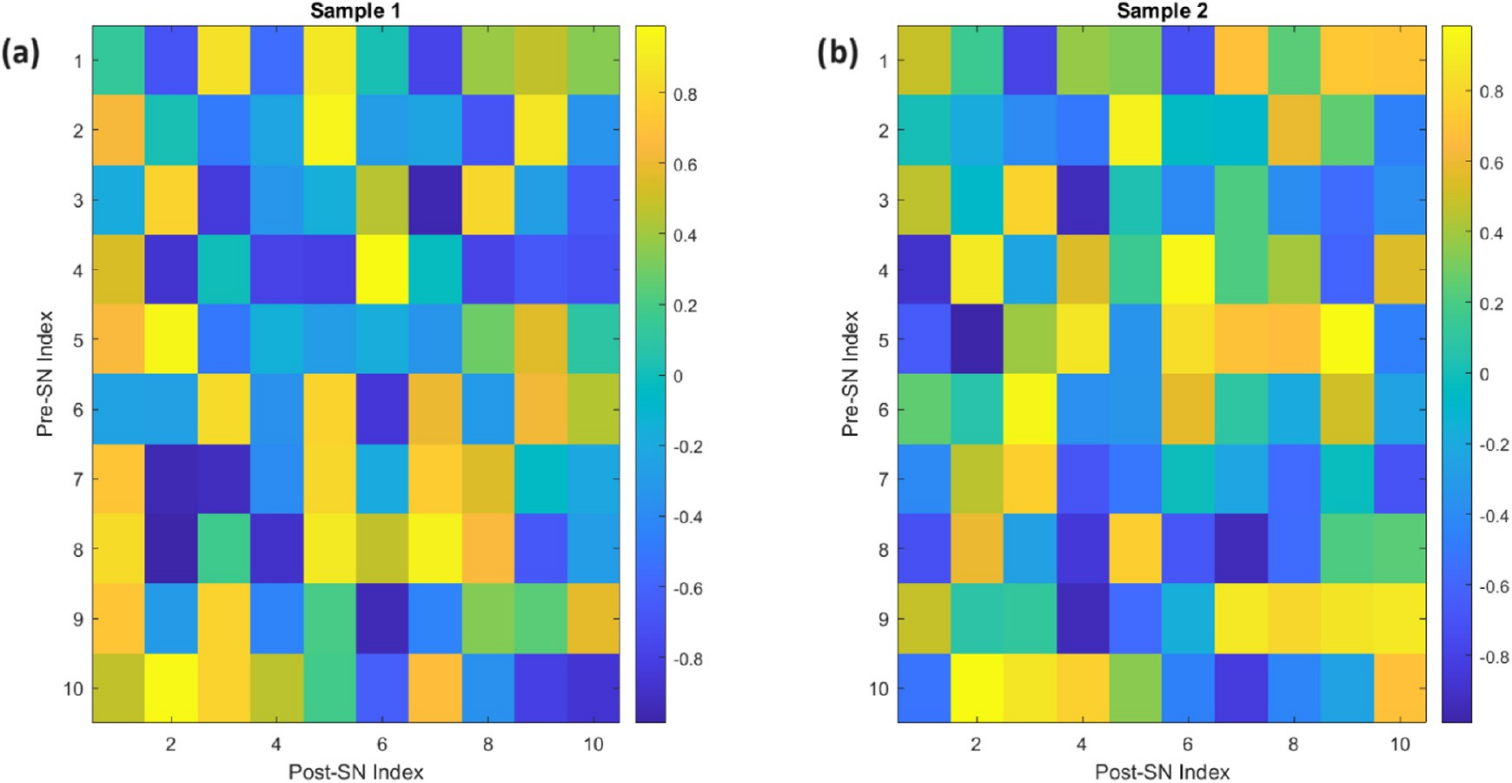
Visualizing the initial synaptic weights in Temporal Coding Neural Networks that were trained using Kombucha-Proteinoid samples. Figure (a) displays the heatmap representation of the initial synaptic weights for Sample 1. This sample is the Kombucha–Proteinoid blend, which has a composition of 40% Kombucha and 60% Proteinoid, measured in volume. The heatmap facilitates a clear comprehension of the connection pattern between the presynaptic and postsynaptic neurons in the network. The heatmap in Figure (b) displays the initial synaptic weights for Sample 2, representing the composition ratio of Kombucha–Proteinoid as 25%:75% (v/v). This heatmap offers a clear depiction of the network structure involved in a diverse composition scenario. The plots utilize a color gradient to represent the synaptic connections’ strength, where warmer colors suggest stronger inter–neuronal communication. These heatmaps provide vital insights into the structure and arrangement of the neural network for various mixtures of Kombucha–Proteinoid proto–brains.

According to Mougkogiannis et al., let $t\in R^{n}$ represent a vector of time values measured in seconds, $p\in R^{n\times 2}$ represent a matrix of potential values measured in volts for 2 different samples.^60^ N∈N denotes the number of neurons in the Neural Network (NN), $T\in R^{+}$ indicates the time window for temporal coding measured in seconds, and $\theta \in R^{+}$ represents the threshold for spike detection measured in volts. Matrices *c* ∈ {0, 1}^*N* × *n*^ and *W* ∈ [−1, 1]^*N* × *N*^ symbolize the temporal codes for each neuron over time and the synaptic weights between neurons, respectively.

For each sample, denoted by *i* = 1, ···, 2, the temporal code *c*_*j*,*i*_ will be equal to 1 if the corresponding potential value *p*_*j*,*i*_ exceeds the threshold θ, and it will be multiplied by the time duration (*T* – min_*k*∈[1,*n*]_{*p*_*k*,*i*_: *p*_*k*,*i*_ > θ}). The indicator function 1_*p*_*j*,*i*_>θ_ returns 1 if the potential value *p*_*j*,*i*_ is greater than θ, and 0 otherwise.

The neurons in the neural network are differentiated by their unique temporal coding, input parameters, and synaptic weights. When the temporal code (denoted as *c*_:,*j*_) surpasses the threshold parameter (θ), the neuron produces an action potential similar to that of a real neuron, transmitting signals through its axonal connections. The proteinoid neurons bear a resemblance to the neurons in a biological nervous system. The synaptic weights (*W*) in the neural network control the strength of the connections between axons and dendrites, serving a similar function to synapses in natural neural systems.

## Discussion

Kombucha–Proteinoid (KP) living electronics signify a significant advancement within the rapidly developing domain of biohybrid technologies, which involve the integration of organic constituents into conventional computational systems. Our findings indicate that the integration of these biotic and abiotic materials in a synergistic manner generates emergent functionality, which in turn stimulates innovation in a wide range of technological fields. Most notably, KP electronics demonstrate the intricate information processing capabilities that are essential for supplying power to adaptive robotics inspired by biology. KP devices have the potential to function as sensors that enable robots to react to environmental stimuli, in addition to circuits that facilitate advanced control systems that govern realistic movement and behavior. These organic electronics would endow these devices with a capability comparable to that of cells, enabling them to perceive and adapt to their environment. Furthermore, the distinctive electrochemical characteristics of KP have the potential to significantly enhance synthetic biomedical apparatus by providing biocompatible interfaces for prosthetics. In addition, lab-on-a-chip devices could incorporate programmable KP sensors and microfluidic scaffolds to capture physiological dynamics, thereby transforming drug testing and disease diagnosis. Beyond the realm of biomedicine, the engineered protein nanostructures and exceptional electrical signaling behaviors that emerge from KP offer a flexible foundation for advancements in chemical synthesis, green electronics, and bioimaging. Simulations of organs and tissues, biodegradable cables, circuits, and computer chips–the scope of possibilities is astounding. Obviously, our exploration of the potential applications for biohybrid KP electronics is far from exhaustive. However, the vast array of functionalities that have been demonstrated already suggests that these living materials have the capacity to revolutionize technology by fusing the most advantageous aspects of the natural and synthetic realms.^61–63^

- How can we design intelligent systems that imitate the information processing and adaptive behaviors of natural cognition?

Robust information processing, adaptation, and emergent cognition are enabled by the integration of heterogeneous components in synthetic bio–computational systems. Proteinoids and Kombucha each bring their own unique set of properties that, when combined, form a foundation for advanced logic application and learning, as seen in Table 1. In particular, proteinoids operate as reconfigurable logic gates when they self-assemble into micronetworks that may carry electrical signals and execute Boolean logic operations (Table 1).^31^ In contrast to logic, which remains constant regardless of inputs, adaptive learning requires constant tuning as inputs change. The unique metabolic pathways of the microbial community in Kombucha are responsible for its reconfigurability (Table 1). Based on our electrical measurements, these pathways exhibit signal processing capabilities that could potentially enable synaptic-like plasticity, though further studies are needed to fully characterize this behavior. Prior work on microbial communities suggests mechanisms similar to long-term potentiation in neural networks.^64,65^ In conclusion, by combining these two biocomponents, complex biologic circuits can be built, equipped with sensory processing capabilities and adaptive programming, and able to mimic important aspects of natural cognition in a computing living platform. Here, bottom-up synthetic biology and unconventional computation come together to show how designed biological systems can become more than the sum of their parts.

- Which complementary biomaterials can be synthesized into circuits capable of information processing and adaptation?
- How can predictive modeling and synthetic biology be integrated to enable robust emergent behaviors in bacteria-based biohybrid systems? For example, we can model these systems using established neural network architectures, such as
13 $$y=f\left(\sum_{i=1}^{n}w_{i}x_{i}+b\right)$$
  Here, *f* is the activation function. The *w*_*i*_ are the connection weights. They measure the strength of bioelectrical coupling between components. The *x*_*i*_ are the input signals from different parts of the bacterial network. *b* is the baseline activity. This would allow a direct comparison with neural networks.^66–69^ It would provide metrics for system performance.

**Table 1:** Comparison of Properties Enabling Computation and Cognition

	Proteinoids	Kombucha	Kombucha–Proteinoids
structure	assemblies and networks	cellulose matrix	embedded composites
signaling properties	electrical spiking	ion conduction	tunable conductivity
information processing	logic operations	synaptic-like	reconfigurable transforms
adaptability	learning	metabolic pathways	morphable networks
emergent cognition	biological neural networks	microbial intelligence	bioinspired computing

A recent study conducted by Leaman et al. presents a convincing illustration of how a data-driven statistical method may effectively describe the movement patterns in two distinct types of Escherichia coli swarms.^70^ Their model, in conjunction with a collaborative gene expression scheme, identified variations in the time frames for planned emergent behaviors among the bacterial groups. The study showcases the effectiveness of combining synthetic biology approaches with computational modeling to enhance engineered biological systems by discovering crucial factors that control their resilience and ability to emerge. As demonstrated in the research conducted by Buss et al., the swift movement and genetic adaptability of E. coli make it very suitable for drug delivery^71^

- What synergistic effects emerge from unifying proteinoids and Kombucha within a synthetic bio-system?

The hybrid system of microbial Kombucha networks interwoven with synthetic proteinoid microspheres shows promise for bioinspired information processing and adaptive computation. The Kombucha SCOBY mat’s cellulose-secreting properties provide a dynamic biotic scaffolding for housing and interfacing the proteinoids. This extracellular structure derived from self-organized bacteria and yeasts provides a living architecture ideal for proteinoid integration, with nutrient diffusion networks and signaling pathways ripe for coupling. Meanwhile, the proteinoids’ customizability down to specific amino acid monomers allows for precise control of new characteristics and computing capacities. Proteinoids, which are polymerized using methods that mimic hypothesized prebiotic chemistry, are programmable protocells that can integrate modular and excitable computing features inside the microbial film ecology. Emergent computational and cognitive system behaviors appear at the interface of bottom–up synthetic biology and top–down living scaffold assembly. The interaction of SCOBY secreted cellulose, electron–transporting aceto–bacteria, spiking proteinoid micro–spheres, and shuttling molecular signals results in a dynamic bio–abiotic network with learning, adaptation, and information transformation characteristics far exceeding those of its isolated constituents. As the study of natural computing expands the substrates and architectures employed for processing, hybrid biosynthetic materials that balance intrinsic microbial intelligence with introducible synthetic block modularity will become increasingly powerful.

A promising use for KP systems is as smart sensors for indirect environmental monitoring.^72–76^ These bioelectronic composites are highly sensitive to external stimuli. Their unique voltage–current characteristics enable a novel sensing method. Rather than needing dedicated sensors for each environmental parameter, KP materials could be multifunctional sensors. Their electrical response patterns would indicate specific conditions. This approach uses the material’s responsiveness to stimuli. These include temperature, pH, chemical species, and mechanical stress. KP-based devices could enable advanced environmental sensing with minimal extra hardware. By studying how the voltage–current response shifts under different conditions. And by using the right machine learning algorithms. This sensing could help in applications needing constant monitoring in complex biological environments.^64,65,75,77,78^ Examples include in vivo medical diagnostics and environmental monitoring. Traditional sensor arrays might be impractical or intrusive. A big advantage is inferring multiple environmental parameters from a single KP substrate’s electrical measurements. It simplifies the system, cuts costs, and allows for miniaturization.

Based on our results—especially the voltage-controlled logic (Figure 8), phase-space dynamics (Figure 9), and the integration of proteinoids in the SCOBY matrix (Table 1)—we propose these key studies:

In order to thoroughly analyze the unconventional computing capabilities of the Kombucha–Proteinoid system, would necessitate several key lines of investigation. Following our observation of basic logic gate behavior, more complex experiments employing electrophysiology may shed light on the synaptic-like plasticity and electrical signaling characteristics of KP networks, thereby identifying bioelectric features that are conducive to learning and computation. Expanding on the observed phase-space patterns, logic implementations and architecture design that make use of the emergent topology may exhibit comparable performance to conventional computing on pattern recognition tasks. Given our successful demonstration of tunable conductivity in the KP composite, the study of information dynamics in KP circuits using biophysical models may yield valuable insights into the optimization of computational capacities. Building on our initial logic gate characterizations, by applying bilateral information and entropy metrics to the analysis of KP logic gate experiments, the extent of information processing in relation to biological cognition may be revealed. Furthermore, taking advantage of our understanding of the KP interface demonstrated in this work, an investigation into the impacts of electrical, topological, and biochemical tuning protocols may reveal techniques for training substrates for high–performance KP computation. A thorough examination of the unconventional computing capabilities of the binary component Kombucha–Proteinoid system requires a multidisciplinary approach encompassing electrophysiology, bio–inspired engineering, biophysics, and information theory. Further effort is required to optimize KP composites for specialized applications; however, the underlying opportunity is evident: follow nature rather than resisting it.

- What further domains might benefit from this fusion of microbial scaffolds and synthetic biology?

The emergent conductive behaviors demonstrated by merging synthetic proteinoids with living Kombucha microbial scaffolds, as investigated in this study, demonstrate the vast potential of biohybrid materials for unconventional information processing. However, as illustrated in Figure 11, these bioelectronic devices represent only a small subset of the options available in the developing field of natural computing engineering. Myriad organic substrates, ranging from organisms such as lichen fungi to self-assembled structures such as cytoskeletal filaments and artificially crafted components such as DNA-based logic gates, all provide fertile ground for the implementation of alternative modes of data representation, memory encoding, and computational operations.

**Figure 11:**
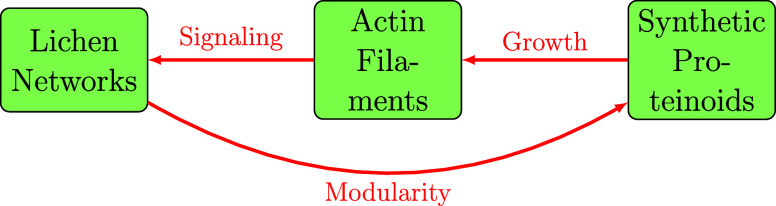
Substrates inspired by biological systems for the purpose of bio–computation. Organisms, natural structures, and synthetic analogues, such as lichen Networks, actin filaments, and synthetic proteinoids, can be used as initial models for developing unconventional information processing methods in engineering. Lichens are symbiotic organisms. They combine fungi with algae or cyanobacteria. They form complex networks. The fungal partner provides structure and protection. The photosynthetic partner supplies nutrients. This natural cooperation is a model for distributed information processing.^79–82^ Within these systems, Lichen Networks employ signaling routes to provide efficient communication and information exchange among many components. Actin filaments serve as a flexible structure that facilitates development and reorganization, allowing for adaptation and responsiveness to stimuli. Synthetic proteinoids demonstrate emergent characteristics, including modularity and self-assembly, as evidenced by their electrical spiking behavior, network formation capabilities, and reconfigurable logic operations demonstrated in this study, that can be utilized for unconventional computing purposes. The interaction between signaling, growth, and modularity in these systems allows for the development of new types of biocomputation, which can be applied in areas such as biological intelligence and bioinformatics.

## Conclusions

Kombucha–proteinoid composite biofilms represent an innovative approach to developing bioelectronic materials. This study shows that we can integrate synthetic proteinoids into a bacterial cellulose matrix. It creates a stable biointerface between the two components. Our experiments show that these composite films can process electrical signals. This work sets the stage for their use in bioelectronics. Furthermore, the material’s inherent sensitivity to environmental conditions, coupled with its unique electrical response patterns, demonstrates significant potential for intelligent sensing applications. These films need further development to unlock functions like unconventional computation. But their electrical properties and stability are promising for future research. The ability to modify the emergent electronic properties of the proteinoids and adjust their composition within the zoogleal nanostructure underscores the potential for customization of these biohybrid films to suit a wide range of applications. Beyond particular functionalities, the bioinspired approach integrates synthetic processing and resilience with realistic excitation, growth, and adaptation, which is critical for the development of next-generation technologies that exist at the interface of machines and organisms (Table 2).

**Table 2:** Key Principles and Their Experimental Demonstrations for Intelligent Sensing Applications

principle	demonstration in this study
environmental response	voltage–current characteristics vary with external conditions through the SCOBY matrix interface
multiparameter sensing	simultaneous monitoring of pH, temperature, and ionic changes via single KP composite
signal integration	proteinoid networks process multiple input signals within the cellulose scaffold
adaptive sensing	microbial community metabolic pathways modify sensing response over time
pattern recognition	electrical spiking behavior changes systematically with environmental stimuli

Although additional work is required to completely implement these preliminary ideas into operational systems, particularly in calibrating and validating the indirect sensing capabilities, the principles outlined in this study represent a promising advancement toward future integrated bio-abio systems based on the fundamental concepts of biological intelligence, organization, and adaptability. The convergence of the technological advancements of humanity and the accomplishments of nature spanning billions of years continues to unveil profound scientific and engineering prospects.

## Methods

### Fabrication Process for Kombucha–Proteinoid Composites

Kombucha–Proteinoid mixture is synthesized by integrating proteinoids within Kombucha cellulose pellicles. Kombucha films are first grown by infusing tea and sugar with boiled water, then inoculating with a SCOBY symbiotic culture and incubating at 20–23 °C in darkness. Once a cellulosic biofilm forms, proteinoids are introduced by injecting proteinoid solutions into the mat and applying proteinoids to the surface. The composite is returned to the incubator for 24 h to allow proteinoid diffusion and attachment (Figure 12). Kombucha provides a robust scaffold with rapid growth, while proteinoids confer tailored functionality like signaling dynamics. Resulting composites exhibit periodic electrical activity reminiscent of spiking neurons. By pairing Kombucha’s structural qualities with proteinoids’ excitability, adaptable bioelectronic materials are produced. Their biotic–abiotic hybrid nature enables emergent capabilities. Studying factors influencing the integration process, including culture conditions, materials ratios, and assembly kinetics, will enable optimization of mixture synthesis. Figure 13a depicts the modular biofabrication process, which starts with the cultivation of the Kombucha cellulose matrix. The hydrogel film demonstrates valuable characteristics such as fast growth, tunable scalability, and basic electrical conduction before the proteinoid is introduced. The introduction of proteinoids is facilitated through injection methods and surface attachment, enabling diffusion and interaction with the conductive scaffold (Figure 13b). The figures clearly demonstrate the successful development of controlled Kombucha culture and the infusion of programmed proteinoids, allowing for the engineering of composite materials with specific characteristics. Continued refinement of growth conditions, culture durations, proteinoid compositions, and assembly kinetics is enhancing the precise integration of components in these hybrid living materials.

**Figure 12:**
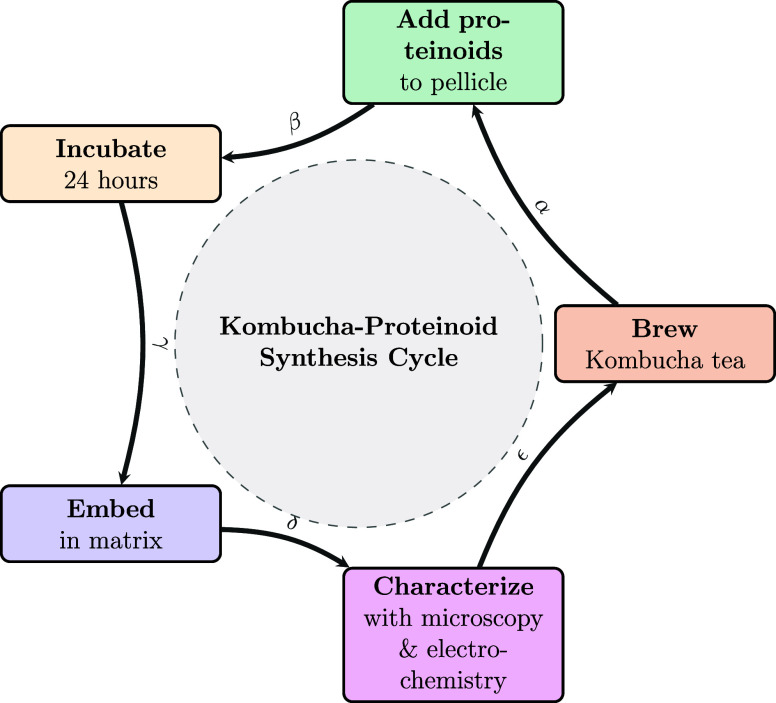
Kombucha–Proteinoid sample is synthesized through a five-step process. A dynamic structure is produced where yeasts and bacteria coexist within cellulose fibers. Proteinoid microspheres are inserted into the tiny structure and attach to the biofilm. After incubating for 1 day, the proteinoids get embedded within the gelatinous microbial mesh, allowing for integration. Ultimately, composites undergo analysis using microscopes and voltmeters, which unveil neural-like signaling patterns. Cycling arrows denote iterative refinement of preparation protocols.

**Figure 13:**
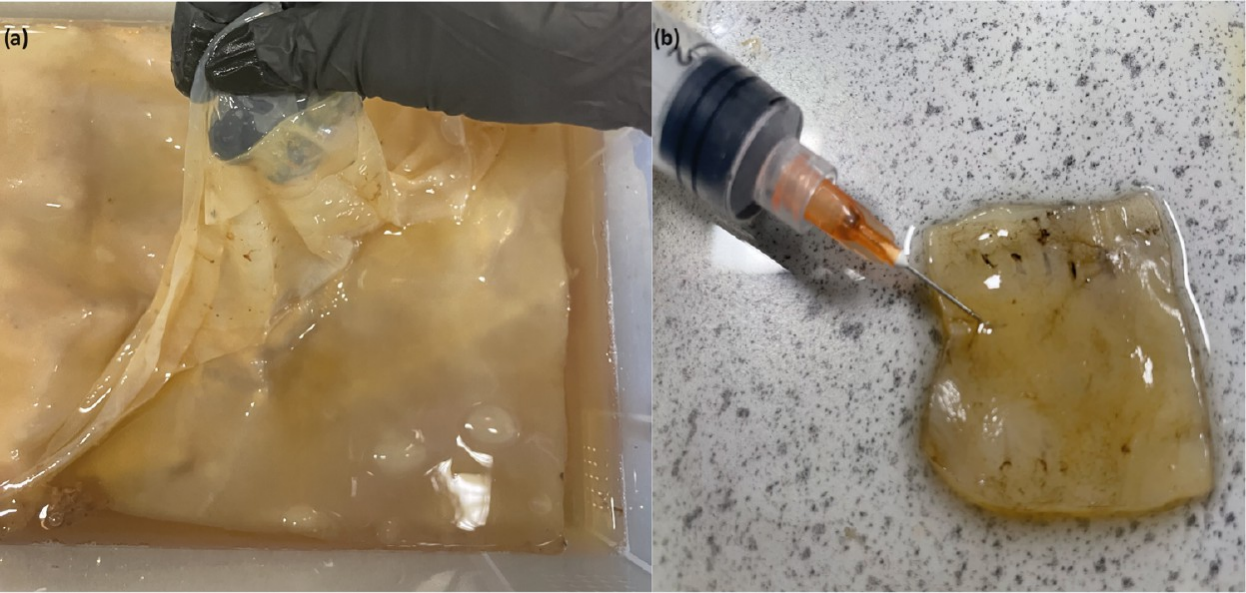
Formation of Kombucha–proteinoid composites. (a) Cultivation of Kombucha cellulose matrix prior to proteinoid integration. (b) Introduction of proteinoids into the mature Kombucha biofilm through injection and surface application techniques.

### Electrical Measurement and Analysis

The experiment used a BK Precision 4053 MHz dual-channel waveform generator to generate the electrical stimuli. To transmit the impulses and record the responses, platinum–iridium electrodes with a diameter of 0.2 mm and a spacing of 10 mm were inserted 5 mm deep into the KP solutions. Before each measurement, electrodes were cleaned with isopropyl alcohol and rinsed with deionized water to ensure consistent surface conditions. Data acquisition was performed using multiple instruments. A Rigol oscilloscope (2 Channel, 100 MHz to 1 GSa/s) was used for real-time monitoring of high-frequency responses. The sampling rate was set to 1 GSa/s for capturing fast transients, with the timebase varied from 1 μs/div to 1 s/div depending on the input signal frequency. A PicoLog ADC-24 was employed for long-term data logging of slow varying signals. This 24-bit resolution device was typically set to a sampling rate of 1 Hz. Additionally, a Picoscope was utilized for detailed waveform analysis and frequency domain measurements, including 4096-point FFT for spectral analysis of the output signals. *I*–*V* characteristics were measured using a Keithley 2450 sourcemeter. Voltage sweeps were performed from −5 to +5 V with a step size of 0.1 V and a sweep rate of 0.1 V/s to minimize capacitive effects. The current measurement range was automatically adjusted from 10 nA to 1 mA full scale. Environmental conditions were controlled, with temperature maintained at 25 °C ± 1 °C using a thermostatically controlled enclosure, and humidity kept at 50% ± 5% RH. All measurements were performed in a Faraday cage to minimize external electromagnetic interference. The data was processed and analyzed using MATLAB R2021a, utilizing customized scripts for noise filtration, baseline adjustment, and feature extraction. The statistical analysis was performed built-in MATLAB functions. In order to ensure repeatability, we have taken measurements over a period of several hours for each recording. A minimum of three repetitions were conducted for every measurement. Validation of the measuring setup was done through control experiments using ordinary resistors and capacitors.

## Notes

**Data Availability** The data sets generated and/or analyzed during the current study are available in the zenodo repository, [https://zenodo.org/records/10206807].

## References

[1] Dimidi E., Cox S. R., Rossi M., Whelan K. (2019). Fermented foods: definitions
and characteristics, impact on the gut microbiota and effects on gastrointestinal
health and disease. Nutrients.

[2] Tietze, H. Kombucha: The Miracle Fungus; Harald Tietze Publishing P/, 1996.

[3] Greenwalt C., Steinkraus K., Ledford R. (2000). Kombucha, the fermented tea: microbiology,
composition, and claimed health effects. J.
Food Prot..

[4] dos Santos, M. J. Kombucha: Caracterização da Microbiota e Desenvolvimento de Novos Produtos Alimentares Para Uso em Restauração, Ph.D. Thesis; Universidade NOVA de Lisboa, 2016.

[5] Coelho R. M.
D., de Almeida A. L., do Amaral R. Q. G., da Mota R. N., de Sousa P. H. M. (2020). Kombucha: Review. Int. J. Gastronomy Food Sci..

[6] Mougkogiannis P., Nikolaidou A., Adamatzky A. (2025). Living electronics
in cellulose zoogleal
mats. Carbohydr. Polym. Technol. Appl..

[7] Jayabalan, R. ; Malbasa, R. ; Sathishkumar, M. ; Kombucha Kombucha. In Reference Module in Food Science; Elsevier, 2016; Vol. 11, p 422.

[8] Frank, G. W. Kombucha: Healthy Beverage and Natural Remedy from the Far East; Its Correct Preparation and Use; Ennsthaler, 1991.

[9] Fu C., Yan F., Cao Z., Xie F., Lin J. (2014). Antioxidant activities
of kombucha prepared from three different substrates and changes in
content of probiotics during storage. Food Sci.
Technol..

[10] Gaggìa F., Baffoni L., Galiano M., Nielsen D. S., Jakobsen R. R., Castro-Mejía J. L., Bosi S., Truzzi F., Musumeci F., Dinelli G., Di Gioia D. (2019). Kombucha beverage from
green, black and rooibos teas: A comparative study looking at microbiology,
chemistry and antioxidant activity. Nutrients.

[11] Lončar E., Djurić M., Malbaša R., Kolarov L., Klašnja M. (2006). Influence
of working conditions upon kombucha conducted fermentation of black
tea. Food Bioprod. Process..

[12] Chen C., Liu B. (2000). Changes in major components
of tea fungus metabolites during prolonged
fermentation. J. Appl. Microbiol..

[13] De
Filippis F., Troise A. D., Vitaglione P., Ercolini D. (2018). Different temperatures select distinctive acetic acid
bacteria species and promotes organic acids production during Kombucha
tea fermentation. Food Microbiol..

[14] Fox S. W. (1995). Thermal
synthesis of amino acids and the origin of life. Geochim. Cosmochim. Acta.

[15] Fox S. W., Harada K. (1960). The thermal copolymerization of amino
acids common
to protein1. J. Am. Chem. Soc..

[16] Dose K. (1974). Chemical and
catalytical properties of thermal polymers of amino acids (proteinoids). Origins Life.

[17] Melius, P. ; Nicolaou, V. Molecular Evolution and Protobiology; Springer, 1984; pp 125–132.

[18] Mougkogiannis, P. ; Adamatzky, A. Morphologies of proteinoids ChemRxiv, 2023.

[19] Mougkogiannis P., Adamatzky A. (2023). Proteinoid
microspheres as protoneural networks. ACS Omega.

[20] Mougkogiannis P., Adamatzky A. (2024). Memfractance
of Proteinoids. ACS Omega.

[21] Mougkogiannis P., Adamatzky A. (2023). Low frequency
electrical waves in ensembles of proteinoid
microspheres. Sci. Rep..

[22] Mougkogiannis P., Kheirabadi N. R., Chiolerio A., Adamatzky A. (2023). Electrical
spiking activity of proteinoids-ZnO colloids. bioRxiv.

[23] Fox, S. W. Evolution of Information Processing Systems: An Interdisciplinary Approach for a New Understanding of Nature and Society; Springer, 1992; pp 203–228.

[24] Mougkogiannis P., Adamatzky A. (2024). Proto-Neurons
from Abiotic Polypeptides. Encyclopedia.

[25] Przybylski A. T., Fox S. W. (1984). Excitable artificial cells of proteinoid. Appl. Biochem. Biotechnol..

[26] Wolman Y., Haverland W. J., Miller S. L. (1972). Nonprotein amino acids from spark
discharges and their comparison with the Murchison meteorite amino
acids. Proc. Natl. Acad. Sci. U.S.A..

[27] Przybylski A. T., Stratten W. P., Syren R. M., Fox S. W. (1982). Membrane, action,
and oscillatory potentials in simulated protocells. Naturwissenschaften.

[28] Fox, S. W. ; Dose, K. Molecular evolution and the origin of life. 1977.

[29] Mougkogiannis P., Adamatzky A. (2024). Proto-Neurons from Abiotic Polypeptides. Encyclopedia.

[30] Fox S. W., Jungck J. R., Nakashima T. (1974). From proteinoid
microsphere to contemporary
cell: formation of internucleotide and peptide bonds by proteinoid
particles. Origins Life.

[31] Mougkogiannis P., Adamatzky A. (2023). Light-induced
spiking in proteinoids yields Boolean
gates. Mater. Des..

[32] Adamatzky A. (2021). Towards proteinoid
computers. Hypothesis paper. Biosystems.

[33] Hsu, L. H.-N. L. Proteinoid Microspheres As Models For Primordial Organic Evolving Systems, Ph.D. Thesis; University of Miami, 1974.

[34] Matsuno, K. Molecular Evolution and Protobiology; Springer Science & Business Media, 2012.

[35] Adamatzky A. (2023). Electrical
potential spiking of kombucha zoogleal mats: A symbiotic community
of bacteria and yeasts. Bioelectricity.

[36] Mougkogiannis P., Adamatzky A. (2023). Logical gates
in ensembles of proteinoid microspheres. PLoS
One.

[37] Mougkogiannis P., Adamatzky A. (2023). Recognition
of sounds by ensembles of proteinoids. bioRxiv.

[38] Piro B., Tran H. V., Thu V. T. (2020). Sensors
Made of Natural Renewable
Materials: Efficiency, Recyclability or Biodegradability–The
Green Electronics. Sensors.

[39] Cao Y., Uhrich K. E. (2019). Biodegradable and
biocompatible polymers for electronic
applications: A review. J. Bioact. Compat. Polym..

[40] Borghetti J., Snider G. S., Kuekes P. J., Yang J. J., Stewart D. R., Williams R. S. (2010). ’Memristive’switches
enable ’stateful’logic
operations via material implication. Nature.

[41] Beasley A. E., Abdelouahab M.-S., Lozi R., Tsompanas M.-A., Powell A. L., Adamatzky A. (2021). Mem-fractive
properties of mushrooms. Bioinspiration Biomimetics.

[42] Adamatzky A., Tarabella G., Phillips N., Chiolerio A., D’Angelo P., Nikolaidou A., Sirakoulis G. C. (2023). Kombucha
electronics: electronic circuits on kombucha mats. Sci. Rep..

[43] Adamatzky, A. Electrical potential spiking of kombucha zoogleal mats bioRxiv 2022, 10.1101/2022.08.03.502684.

[44] Strukov D. B., Snider G. S., Stewart D. R., Williams R. S. (2008). The missing memristor
found. Nature.

[45] Wang Z., Joshi S., Savel’ev S. E., Jiang H., Midya R., Lin P., Hu M., Ge N., Strachan J. P., Li Z. (2017). Memristors with diffusive
dynamics as synaptic emulators for neuromorphic
computing. Nat. Mater..

[46] Pershin Y. V., Di Ventra M. (2019). A simple test
for ideal memristors. J. Phys. D: Appl. Phys..

[47] Mellor, C. HP 100TB Memristor Drives by 2018—If You’re Lucky, Admits Tech Titan, 2013. https://www.theregister.com/2013/11/01/hp_memristor_2018/.

[48] Williams R. S. (2008). How we
found the missing memristor. IEEE Spectrum.

[49] Meuffels, P. ; Soni, R. Fundamental issues and problems in the realization of memristors, arXiv:1207.7319. arXiv.org e-Print archive, 2012 https://arxiv.org/abs/1207.7319.

[50] Di
Ventra M., Pershin Y. V. (2013). On the physical properties of memristive,
memcapacitive and meminductive systems. Nanotechnology.

[51] Kim J., Pershin Y. V., Yin M., Datta T., Di Ventra M. (2020). An Experimental
Proof that Resistance-Switching Memory Cells are not Memristors. Adv. Electron. Mater..

[52] Sundqvist K. M., Ferry D. K., Kish L. B. (2017). Memristor
equations: Incomplete physics
and undefined passivity/activity. Fluctuation
Noise Lett..

[53] Abraham I. (2018). The case for
rejecting the memristor as a fundamental circuit element. Sci. Rep..

[54] Chua L. (1971). Memristor-the
missing circuit element. IEEE Trans. Circuit
Theory.

[55] Chua L. O., Kang S. M. (1976). Memristive devices and systems. Proc. IEEE.

[56] Mostert A. B., Powell B. J., Gentle I. R., Meredith P. (2012). On the origin of electrical
conductivity in the bio-electronic material melanin. Appl. Phys. Lett..

[57] Adamatzky A. (2022). Dynamics of
electrical resistance of kombucha zoogleal mats. Biophys. Rev. Lett..

[58] Tapias Y. A. R., Peltzer M. A., Delgado J. F., Salvay A. G. (2020). Kombucha
tea by-product
as source of novel materials: Formulation and characterization of
films. Food Bioprocess Technol..

[59] Hausdorff (Box-Counting) Fractal Dimension, 2013. https://uk.mathworks.com/matlabcentral/fileexchange/30329-hausdorff-box-counting-fractal-dimension.

[60] Mougkogiannis P., Adamatzky A. (2023). Proteinoid
Microspheres as Protoneural Networks. ACS Omega.

[61] Lin Z., Jiang T., Shang J. (2022). The emerging
technology of biohybrid
micro-robots: a review. Bio-Des. Manuf..

[62] Menciassi A., Takeuchi S., Kamm R. D. (2020). Biohybrid
systems: Borrowing from
nature to make better machines. APL Bioeng..

[63] Patino T., Mestre R., Sanchez S. (2016). Miniaturized soft bio-hybrid robotics:
a step forward into healthcare applications. Lab Chip.

[64] Shong J., Diaz M. R. J., Collins C. H. (2012). Towards
synthetic microbial consortia
for bioprocessing. Curr. Opin. Biotechnol..

[65] Tagkopoulos I., Liu Y.-C., Tavazoie S. (2008). Predictive
behavior within microbial
genetic networks. Science.

[66] Larsen P. E., Field D., Gilbert J. A. (2012). Predicting
bacterial community assemblages
using an artificial neural network approach. Nat. Methods.

[67] Silva K. P.
T., Boedicker J. Q. (2019). A neural
network model predicts community-level signaling
states in a diverse microbial community. PLoS
Comput. Biol..

[68] Armitage J. P., Holland I. B., Jenal U., Kenny B. (2005). “Neural
networks”
in bacteria: making connections. J. Bacteriol..

[69] Lee J.-Y., Sadler N. C., Egbert R. G., Anderton C. R., Hofmockel K. S., Jansson J. K., Song H.-S. (2020). Deep learning
predicts microbial
interactions from self-organized spatiotemporal patterns. Comput. Struct. Biotechnol. J..

[70] Leaman E. J., Sahari A., Traore M. A., Geuther B. Q., Morrow C. M., Behkam B. (2020). Data-driven statistical
modeling of the emergent behavior
of biohybrid microrobots. APL Bioeng..

[71] Buss N., Yasa O., Alapan Y., Akolpoglu M. B., Sitti M. (2020). Nanoerythrosome-functionalized biohybrid microswimmers. APL Bioeng..

[72] Noble R. R., Stalker L., Wakelin S. A., Pejcic B., Leybourne M. I., Hortle A. L., Michael K. (2012). Biological
monitoring for carbon
capture and storage-a review and potential future developments. Int. J. Greenhouse Gas Control.

[73] Hodge R. A., Longo J. J. (2002). International monitoring
for environmental health surveillance. Can.
J. Public Health.

[74] Tredoux G., Cavé L., Engelbrecht P. (2004). Groundwater pollution: Are we monitoring
appropriate parameters?. Water SA.

[75] Lindenmayer D. B., Likens G. E. (2010). The science and application of ecological monitoring. Biol. Conserv..

[76] Agarwal E., Agarwal R., Garg R. D., Garg P. K. (2013). Delineation
of groundwater
potential zone: An AHP/ANP approach. J. Earth
Syst. Sci..

[77] Cardinale S., Arkin A. P. (2012). Contextualizing context for synthetic biology-identifying
causes of failure of synthetic biological systems. Biotechnol. J..

[78] Bergelson J., Kreitman M., Petrov D. A., Sanchez A., Tikhonov M. (2021). Functional
biology in its natural context: A search for emergent simplicity. eLife.

[79] Adamatzky A., Ayres P., Beasley A. E., Roberts N., Wösten H. A. (2022). Logics
in fungal mycelium networks. Log. Univers..

[80] Betzel R. F., Medaglia J. D., Bassett D. S. (2018). Diversity
of meso-scale architecture
in human and non-human connectomes. Nat. Commun..

[81] Japyassú H. F., Laland K. N. (2017). Extended spider cognition. Anim.
Cognit..

[82] Hollan J., Hutchins E., Kirsh D. (2000). Distributed cognition: toward a new
foundation for human-computer interaction research. ACM Trans. Comput.-Human Interact..

